# Structure, sequon recognition and mechanism of tryptophan *C*-mannosyltransferase

**DOI:** 10.1038/s41589-022-01219-9

**Published:** 2023-01-05

**Authors:** Joël S. Bloch, Alan John, Runyu Mao, Somnath Mukherjee, Jérémy Boilevin, Rossitza N. Irobalieva, Tamis Darbre, Nichollas E. Scott, Jean-Louis Reymond, Anthony A. Kossiakoff, Ethan D. Goddard-Borger, Kaspar P. Locher

**Affiliations:** 1 Institute of Molecular Biology and Biophysics, ETH Zürich, Zürich, Switzerland; 2 The Walter and Eliza Hall Institute of Medical Research, Parkville, Victoria Australia; 3 Department of Medical Biology, University of Melbourne, Parkville, Victoria Australia; 4 Department of Biochemistry and Molecular Biology, University of Chicago, Chicago, IL USA; 5 Department of Chemistry, Biochemistry and Pharmaceutical Sciences, University of Bern, Bern, Switzerland; 6 Department of Microbiology and Immunology, University of Melbourne at the Peter Doherty Institute for Infection and Immunity, Parkville, Victoria Australia; 7 Present Address: Laboratory of Molecular Neurobiology and Biophysics and Howard Hughes Medical Institute, The Rockefeller University, New York, NY USA

**Keywords:** Structural biology, Glycobiology, Enzyme mechanisms

## Abstract

*C*-linked glycosylation is essential for the trafficking, folding and function of secretory and transmembrane proteins involved in cellular communication processes. The tryptophan *C*-mannosyltransferase (CMT) enzymes that install the modification attach a mannose to the first tryptophan of WxxW/C sequons in nascent polypeptide chains by an unknown mechanism. Here, we report cryogenic-electron microscopy structures of *Caenorhabditis* *elegans* CMT in four key states: apo, acceptor peptide-bound, donor-substrate analog-bound and as a trapped ternary complex with both peptide and a donor-substrate mimic bound. The structures indicate how the *C*-mannosylation sequon is recognized by this CMT and its paralogs, and how sequon binding triggers conformational activation of the donor substrate: a process relevant to all glycosyltransferase C superfamily enzymes. Our structural data further indicate that the CMTs adopt an unprecedented electrophilic aromatic substitution mechanism to enable the C-glycosylation of proteins. These results afford opportunities for understanding human disease and therapeutic targeting of specific CMT paralogs.

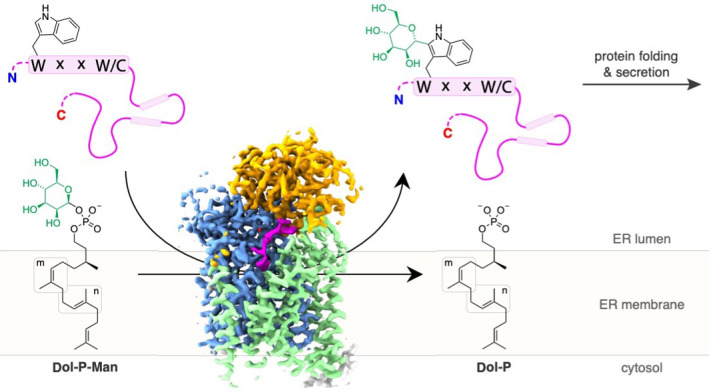

## Abstract (web summary)

Tryptophan *C*-mannosyltransferase (CMT) enzymes append a mannose to the first tryptophan of select sequences, which is important for the trafficking, folding and function of secretory and transmembrane proteins involved in cellular communication processes. A study reveals the structure, mode of peptide recognition and catalytic mechanism for the eukaryotic *C*-mannosyltransferase DPY19.

## Main

**Fig. 1:**
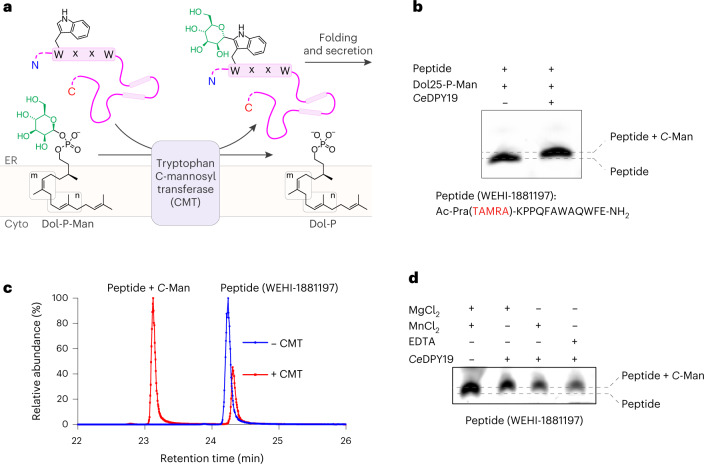
CMT activity is not divalent metal ion dependent. **a**, Schematic of CMT-mediated tryptophan *C*-mannosylation of secretory and transmembrane proteins in the endoplasmic reticulum (ER). Nascent polypeptide chains (pink line) containing the WxxW/C sequon (pink boxes) are mannosylated by CMT using Dol-P-Man (mannosyl group depicted in green) as donor substrate, thereby forming the depicted *C*-glycosidic bond. Glycopeptides are subsequently folded and secreted via the Golgi apparatus. **b**, In vitro *C*-mannosylation reaction using purified CMT *Ce*DPY19. Tricine–SDS–PAGE was used to separate fluorescently labeled acceptor peptide upon mannosylation or unmodified, *n* = 1 independent replicates. **c**, LC–MS analysis of in vitro *C*-mannosylation reaction, demonstrating the attachment of a single hexose to the fluorescently labeled acceptor peptide, *n* = 1 independent replicates. **d**, Tricine–SDS–PAGE analysis of in vitro *C*-mannosylation reaction in presence of the divalent metal ions MnCl_2_ and MgCl_2_ as well as in the absence of divalent metal ions and with *Ce*DPY19 preincubated with the metal ion chelator EDTA, demonstrating that CMT activity is unaffected by the absence of divalent metal ions, *n* = 1 independent replicates. Source data

The posttranslational modification of proteins with carbohydrates includes glycosidic linkages to nitrogen, oxygen and carbon atoms, referred to as *N*-linked, *O*-linked and *C*-linked glycosylation^1^. Among them, *C*-glycosylation is the least-well understood. It involves the formation of a carbon–carbon glycosidic bond between the C2 atom of the tryptophan side chain indole moiety and the anomeric carbon of an α-d-mannopyranose sugar (Fig. 1a)^2^. In humans, approximately 20% of all secreted or transmembrane proteins are predicted to be *C*-mannosylated^3^. The modification has an essential role in the folding^4^, stability^4^, trafficking^5,6^ and function^6,7^ of secretory- and transmembrane proteins^2,6,8^. This includes critical signaling proteins such as the type-I cytokine receptors^9–11^, myelin-associated glycoprotein^8^, adhesion GPCRs^7^ and Wnt/β-catenin^12^. *C*-mannosylation is also found on important pathogen antigens, such as the soluble glycoprotein of Ebola virus^13,14^, and essential adhesins of apicomplexan parasites, including the toxoplasmosis-causing *Toxoplasma gondii*^15^ and malaria-causing *Plasmodium* spp.^16^. Beyond mammalian biology and diseases, *C*-mannosylation has been studied in the model nematode *C. elegans*, where it was shown to control Wnt-signaling to establish left/right asymmetry in neuroblast polarization and migration during development^17^.

While tryptophan *C*-mannosylation was discovered nearly three decades ago^2^, the first tryptophan *C*-mannosyltransferase (CMT) was identified less than a decade ago: the *C. elegans* protein DPY19 (ref. 18). This enzyme was predicted to be an integral membrane protein belonging to the C-type glycosyltransferase superfamily (GT-Cs)^18^. All known CMTs are homologous to the *C. elegans* enzyme (*Ce*DPY19), after which they are named. Unlike *C. elegans*, which contains a single CMT enzyme, humans have four paralogs, termed *Hs*DPY19L1, *Hs*DPY19L2, *Hs*DPY19L3 and *Hs*DPY19L4 (ref. 18), with *Hs*DPY19L1 sharing the greatest sequence similarity with the *C. elegans* homolog^18^. The human paralogs differ in their tissue expression profile and sequon preferences. *Hs*DPY19L1, *Hs*DPY19L3 and *Hs*DPY19L4 are expressed ubiquitously^19^, whereas *Hs*DPY19L2 is found exclusively in the testis^20^, where it plays an essential role in spermatogenesis^21^. Loss of function mutations in *Hs*DPY19L2 induce globozoospermia to cause male infertility^21^.

The reaction catalyzed by CMT enzymes occurs on the luminal side of the endoplasmic reticulum and involves the transfer of a mannose unit from a dolichylphosphate mannose (Dol-P-Man) donor^22^ to an acceptor protein containing a WxxW or WxxC consensus sequon (Fig. 1a)^5^. Like *N*-glycosylation and *O*-mannosylation, tryptophan *C*-mannosylation occurs before or during protein folding^23^.

The true extent and the physiological role of tryptophan *C*-mannosylation has long remained poorly understood. Biochemical tools that allow addressing these questions have only recently been developed. These include synthetic *C*-mannosyl-tryptophan conjugates^24,25^, yeast-based high-throughput in vivo CMT-activity assays^26^, bacterial lectins^27^ and monoclonal antibodies for the enrichment and mass spectrometric analysis of *C*-mannosylated peptides^26^. These tools have been used to map the ‘*C*-glycome’ of the mouse brain^26^ and are driving rapid growth in the field. Yet important unanswered questions remain, including how the CMTs recognize their substrates and catalyze the formation of this unique carbon–carbon bond between protein and carbohydrate.

While the structures and mechanisms of enzyme complexes catalyzing *N*-linked and, in part, *O*-linked protein glycosylation have been described^28–32^, the mechanism of CMT-catalyzed tryptophan *C*-mannosylation is unknown. To understand how CMTs recognize sequons in unfolded proteins and covalently modify acceptor tryptophans with *C*-linked mannose, we determined high-resolution cryogenic-electron microscopy (cryo-EM) structures of the archetypical *C. elegans* enzyme (*Ce*DPY19) in complex with synthetic acceptor and donor substrates to capture distinct states along the reaction coordinate. Structure-guided mutagenesis facilitated a functional analysis of key residues involved in substrate recognition and catalysis. Collectively, these data reveal the basis of CMT substrate specificity and allow a structure-based mechanism to be proposed for this unique glycosylation reaction.

## Results

### CMT activity is not divalent metal ion dependent

**Extended Data Fig. 1:**
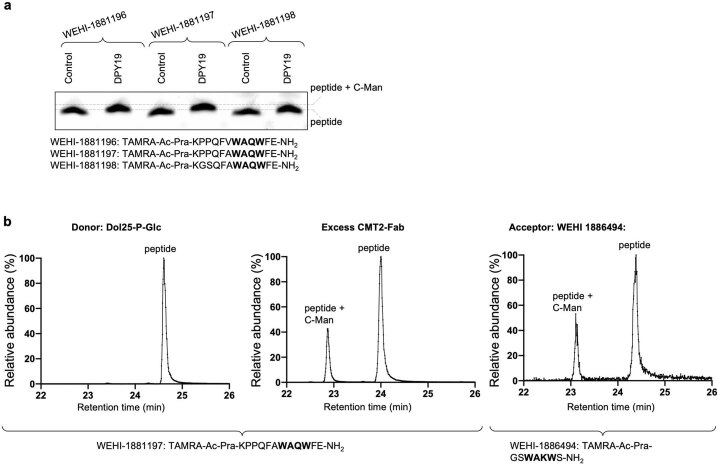
Functional characterization of *Ce*DPY19. **a**, Tricine-SDS-PAGE analysis of *in vitro* activity of purified, detergent reconstituted *Ce*DPY19 for mannosylation of peptides with different sequences, n = 1 independent replicates. **b**, LC-MS analysis of *in vitro* activity of purified, detergent reconstituted *Ce*DPY19 for mannosylation of the peptide WEHI-188197, with Dol25-P-Glc as donor instead of Dol25-P-Man, in presence of CMT2–Fab, and for the peptide WEHI‐1886494, n = 1 independent replicates. Source data

We purified recombinantly expressed *Ce*DPY19 and developed an in vitro assay that recapitulated the tryptophan *C*-mannosylation reaction using synthetic, fluorescently labeled peptides (WEHI-1881196, WEHI-1881197, WEHI-1881198, WEHI-1886494, numbered **1**–**4**) based on known acceptor substrate sequences^2,22^. As a donor substrate, we used a synthetic Dol-P-Man analog (Dol25-P-Man, **5**), a compound that contains five isoprenoid units (25 carbon atoms) and had been successfully used as a mannose donor for the endoplasmic reticulum-resident GT-CMTs ALG3, ALG9 and ALG12 of the *N*-glycosylation pathway^33^. We measured in vitro *C*-mannosylation using Tricine–SDS–PAGE (Fig. 1b and Extended Data Fig. 1a) and liquid chromatography–mass spectrometry (LC–MS) (Fig. 1c), which demonstrated the covalent attachment of a single hexose unit to the peptide. The activity of purified recombinant *Ce*DPY19 was unchanged when reactions were supplemented with Mn^2+^ or Mg^2+^ salts, or with the metal ion chelator EDTA, suggesting that the enzyme does not require divalent metal ion cofactors (Fig. 1d). This is in stark contrast to oligosaccharyltransferase (OST)^34^ and *O*-mannosyltransferase (PMT1/2)^29^, the related GT-C enzymes responsible for *N*- and *O*-glycosylation of proteins: OST activity is metal dependent^34^ and available evidence indicates that PMT1/2 activity is likely also metal dependent^29^. CMT’s metal independence and the unique nature of the chemical bond it forms suggests its mechanism diverges from that of PMT1/2 and OST, despite it using the same donor substrate (Dol-P-Man) as PMT1/2. Finally, we also found that *Ce*DPY19 exclusively processed synthetic Dol-P-Man analogs and did not accept a synthetic, glucose-containing Dol-P-Glc analog (Dol25-P-Glc, **6**)^33^ as a substrate, thus recapitulating the enzyme’s physiological substrate specificity in vitro (Extended Data Fig. 1b).

### Structure and topology of *Ce*DPY19

**Fig. 2:**
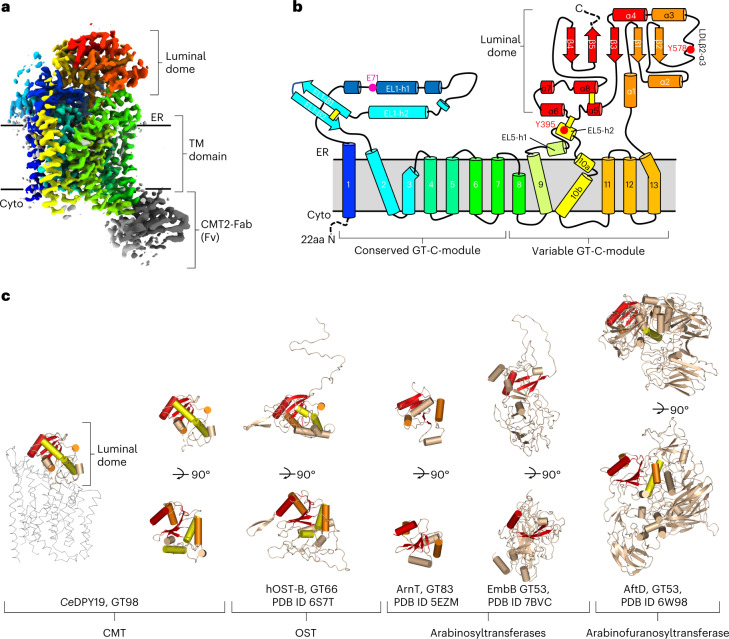
Structure and topology of *Ce*DPY19 and evolutionary conservation of the GT-C ‘luminal domain’ fold. **a**, Cryo-EM map of substrate-free *Ce*DPY19 in rainbow coloring (blue at N terminus, red at C terminus). The Fv portion of the Fab fragment used for cryo-EM studies is colored gray. ER, endoplasmic reticulum. **b**, Schematic representation of *Ce*DPY19 topology, with transmembrane (TM) helices and external loops (EL) numbered and colored as in **a**. A pink sphere indicates the proposed catalytic residue Glu71. Red spheres indicate Tyr395 and Tyr578 that are part of the ‘peptide sensor’ in the active site. Yellow bars indicate disulfide bonds. Regions that were disordered in the structures are indicated with dashed lines. **c**, Structures of the luminal dome of representative GT-C members are aligned to the luminal dome of *Ce*DPY19. The structurally conserved core is highlighted in red, orange and yellow with descending structural conservation.

**Extended Data Fig. 2:**
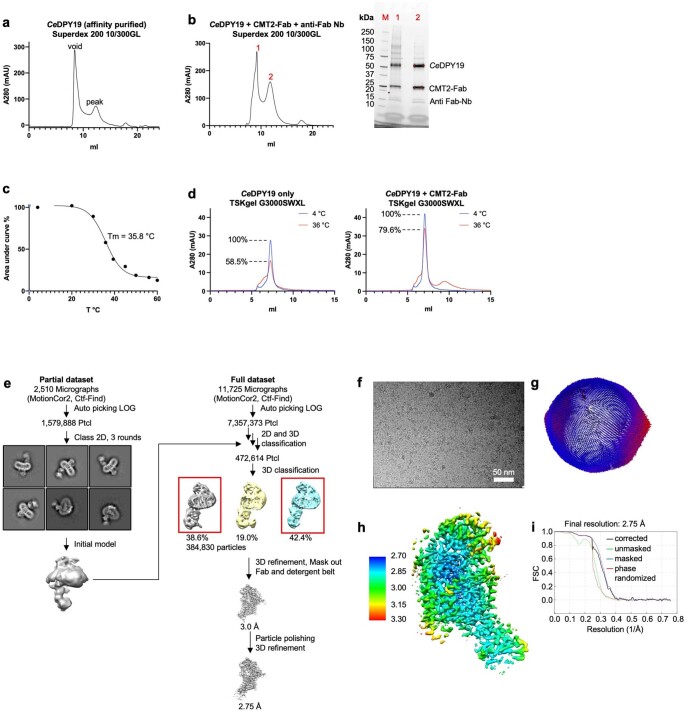
Purification, Fab generation, and structure determination of substrate-free *Ce*DPY19–CMT2-Fab–anti-Fab Nb complex. **a**, Preparative SEC of purified, detergent reconstituted *Ce*DPY19. **b**, Preparative SEC of purified, detergent reconstituted *Ce*DPY19 in complex with CMT2-Fab and anti-Fab nanobody (left) and SDS-PAGE analysis of peak fractions (right). **c**, SEC-TM analysis of purified, detergent reconstituted *Ce*DPY19. A Tm of 35.82 °C was calculated. **d**, Analytical SEC of purified, detergent reconstituted *Ce*DPY19 pre-incubated for 10 min at either 4 °C or at 36 °C in the presence or absence of CMT2-Fab. **e**, Overview of the EM data processing and structure determination pipeline using RELION 3.1. **f**, Representative cryo-EM micrograph. **g**, Spatial distribution of particles in the final iteration of 3D refinement. **h**, Refined, and B-factor sharpened EM map, colored by local resolution estimation as calculated in RELION 3.1. **i**, Resolution estimation of final map via Fourier shell correlation, as calculated in RELION 3.1. Source data

To facilitate high-resolution cryo-EM studies, we used phage display and a synthetic Fab library^35^ and isolated a conformational epitope-binding Fab against *Ce*DPY19 (Extended Data Fig. 2a,b). This approach was shown to increase the size and mass of particles and provide a fiducial mark in particle alignment^33,36^. Among the selected Fabs, we found CMT2-Fab (EC_50_ = 17 nM) to increase the thermostability of the enzyme without affecting the catalytic activity (Extended Data Figs. 1b and 2c,d). We also added an anti-Fab nanobody^37^ that provides additional structural features and thereby breaks the pseudosymmetry of Fab fragments^36^. This approach allowed us to determine a 2.75 Å resolution cryo-EM structure of apo *Ce*DPY19 (Fig. 2a, Extended Data Fig. 2, Supplementary Table 1 and Supplementary Fig. 1).

**Extended Data Fig. 3:**
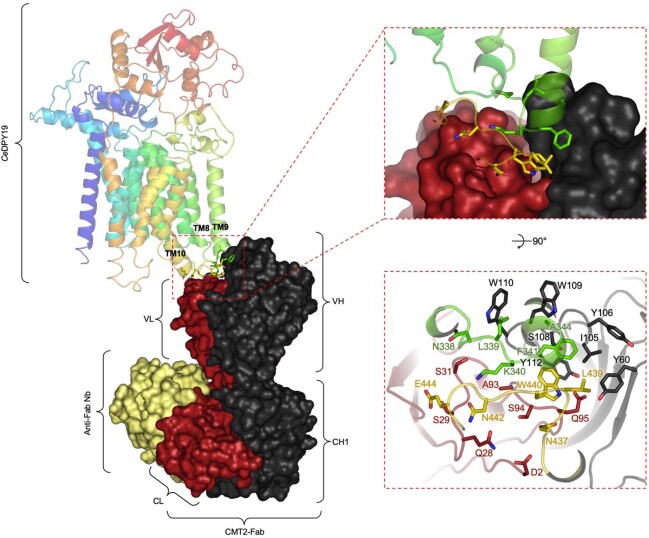
Molecular interactions at the interface of *Ce*DPY19 and CMT2-Fab. The cryo-EM structure of apo-*Ce*DPY19 in complex with CMT2-Fab and anti-Fab Nb is depicted. *Ce*DPY19 is shown in ribbon representation with rainbow coloring, starting in blue at the N-terminus and transitioning to red at the C-terminus. CMT2-Fab is shown in surface representation in black (heavy chain) and red (light chain). The anti-Fab Nb is shown in yellow. The upper insets show a zoomed view of the Fab binding interface with selected residues shown in stick representation.

*Ce*DPY19 contains 13 transmembrane helices, two long endoplasmic reticulum–luminal loops (EL1 and EL5), and a globular, endoplasmic reticulum–luminal, *C*-terminal domain (Fig. 2b). The CMT2-Fab used for these cryo-EM analyses binds to the cytoplasmic loops IL4 and IL5 and is therefore positioned on the opposite side of the membrane, relative to the *Ce*DPY19 active site (Fig. 2a and Supplementary Fig. 3), where it cannot interfere with catalysis (Extended Data Figs. 1b and 3). Like other GT-Cs, *Ce*DPY19 contains a structurally conserved and a variable module^33^. The latter is reminiscent of the variable module of the STT3 subunit of OST^28,30,32^ with respect to transmembrane helix arrangement and fold, suggesting that *C*-linked and *N*-linked protein glycosylation machinery evolved from a common ancestor. The *C*-terminal, endoplasmic reticulum–luminal domain of *Ce*DPY19 contains a core formed by an α_5_β_5_-sandwich (Fig. 2a,b: β1-β5, α1-α4 and α8). As this domain forms a lid-like structure that covers the respective catalytic sites, and given its shape, we refer to it as ‘luminal dome’. On structural alignments, we found the core of the luminal dome to be structurally conserved in otherwise structurally diverse luminal domains of other GT-Cs, including those of OSTs^28,30,32^, bacterial arabinosyltransferases^38,39^ and arabinofuranosyltransferases^40^ (Fig. 2c).

**Extended Data Fig. 4:**
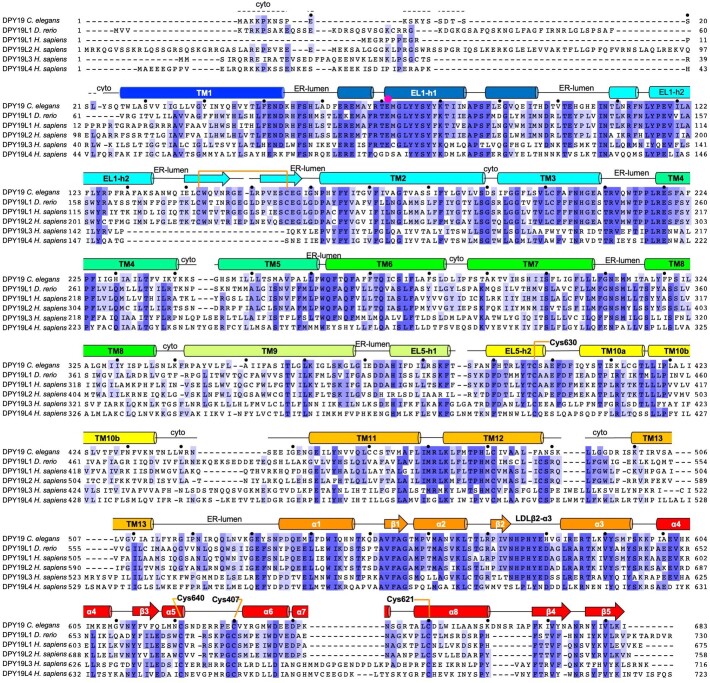
Sequence alignment of CMT homologs and paralogs. Alignment of amino acid sequences of CMT homologs *C. elegans* DPY19, zebrafish DPY19L1, and the human CMT paralogs *Hs*DPY19L1–4, generated with Clustal Omega (Uniprot identifiers: P34413, Q6DRN1, Q2PZI1, Q6NUT2, Q6ZPD9, and Q7Z388). Secondary structure elements of *Ce*DPY19 are depicted and labeled above the sequence. The dashed lines indicate regions that are disordered in the *Ce*DPY19 structures. Cytosolic regions are labeled ‘cyto’ and ER-luminal regions are labeled ‘ER-lumen’. A pink dot indicates the presumed catalytic base Glu71. Black dots indicate every tenth amino acid in the sequence of *Ce*DPY19.

*Ce*DPY19 contains three disulfide bridges. One of them links Cys407 and Cys630, thereby tethering the luminal dome to EL5, the loop connecting transmembrane helices 9 and 10 (Fig. 2b and Extended Data Fig. 4). This is distinct from STT3 in OST, where a reversible engagement and disengagement of EL5 relative to the luminal dome is essential for the binding and release of substrates and products^31^. The presence of this disulfide bond in *Ce*DPY19, which is conserved in CMT enzymes, suggests that substrate binding and product release does not require association and dissociation of these domains.

### Acceptor sequon recognition in unfolded proteins

**Fig. 3:**
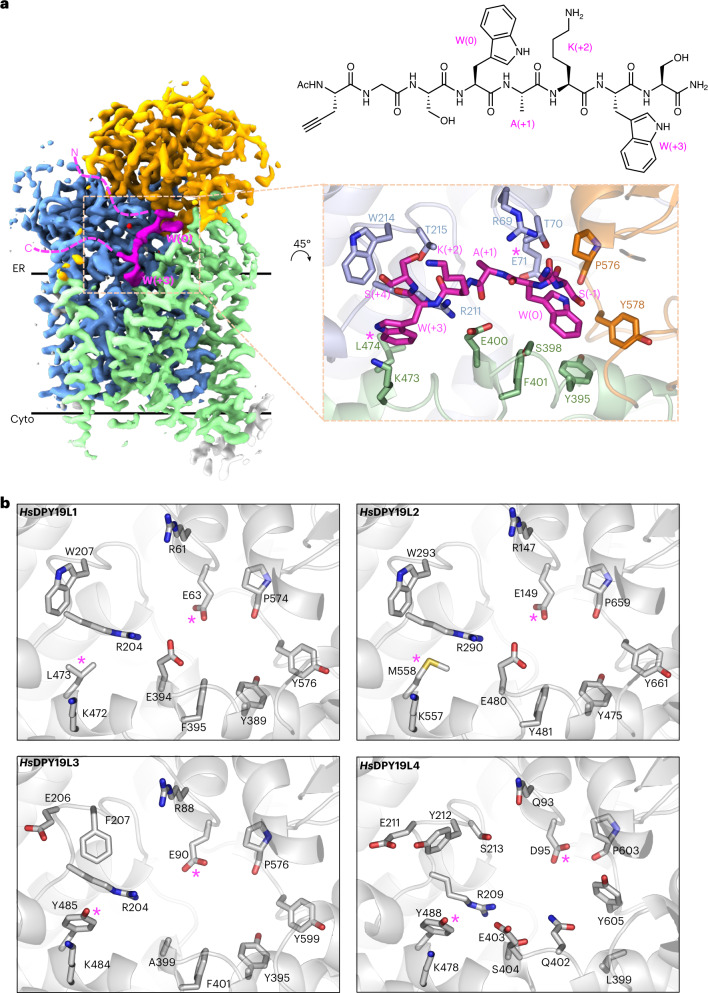
Structural basis of acceptor sequon recognition in unfolded proteins. **a**, Cryo-EM map of peptide-bound *Ce*DPY19 at 2.7 Å resolution. The conserved and variable GT-C modules are colored blue and green, the luminal dome in orange, CMT2-Fab in gray and the bound acceptor peptide in magenta. The locations of the acceptor sequon residues W(0) and W(+3) are labeled and dashed lines illustrate the trajectories of the unfolded N and C terminus of the peptide. The chemical structure of the peptide used for the structure determination of peptide-bound *Ce*DPY19 is shown on the right. The inset shows the binding pocket of the acceptor peptide, with residues in contact with bound substrate shown as sticks and labeled. The locations of the presumed catalytic base Glu71 and the sequon specificity dictating residue Leu474 are indicated by pink asterisks. **b**, Predicted acceptor peptide recognition sites of human CMT-paralog AlphaFold models^42^ *Hs*DPY19L1, *Hs*DPY19L2, *Hs*DPY19L3 and *Hs*DPY19L4 are shown oriented in the same orientation as in **a** (inset), colored white and with the equivalent residues of Glu71 and Leu474 indicated by pink asterisks.

**Fig. 4:**
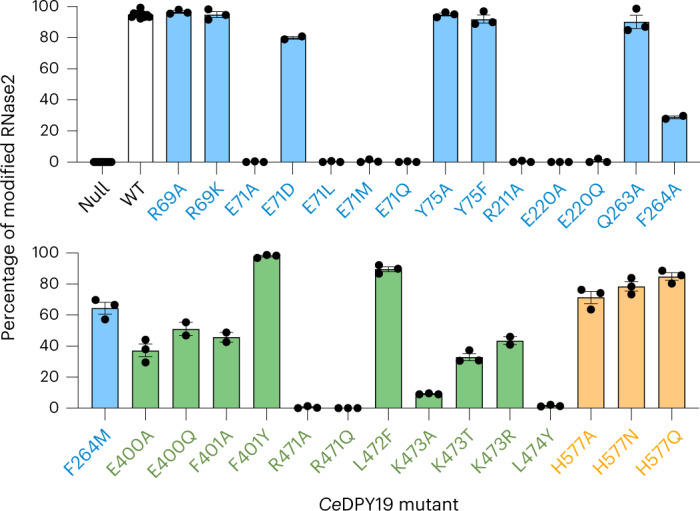
Key residues in CMT-catalyzed tryptophan mannosylation. Normalized occupancy of the sole tryptophan *C*-mannosylation site in human RNase2 when coexpressed with wild type (WT) or mutant *Ce*DPY19 in *P. pastoris*, as determined by LC–MS analysis. Note that *P. pastoris* does not possess any native CMT activity. Normalization is based on the relative abundance of glycosylated and nonglycosylated peptides, as determined by LC–MS with parallel reaction monitoring analyses. Data are presented as mean values ±s.e.m. for *n* = 3 independent replicates. Bars are colored according to the location of the mutation in *Ce*DPY19: blue for residues in the conserved GT-C module, green for residues in the variable GT-C module and orange for residues in the luminal dome. Source data

**Extended Data Fig. 5:**
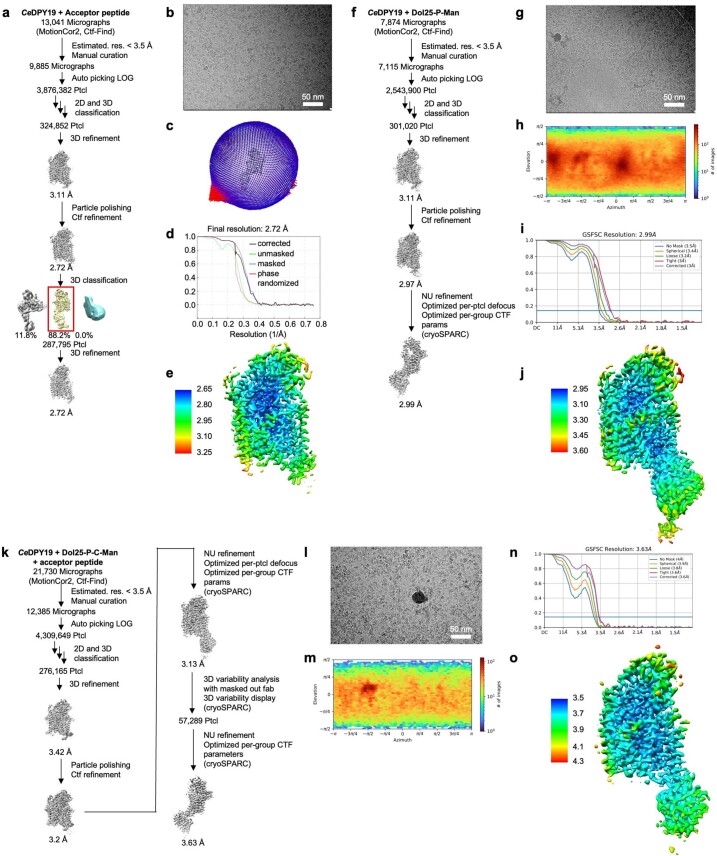
Cryo-EM structure determination of acceptor peptide-bound, donor substrate-bound, and ternary complex of *Ce*DPY19. **a**-**e**, structure determination of acceptor peptide-bound *Ce*DPY19–CMT2-Fab–anti-Fab Nb complex. **a**, Overview of the EM data processing and structure determination pipeline using RELION 3.1 and, if indicated, cryoSPARC v3.2. **b**, Representative cryo-EM micrograph. **c**, Spatial distribution of particles in the final iteration of 3D refinement. **d**, Resolution estimation of final map via Fourier shell correlation, as calculated in cryoSPARC v3.2. **e**, Resolution estimation of final map via Fourier shell correlation, as calculated in RELION 3.1. **f**-**j**, structure determination of Dol_25_-P-Man-bound *Ce*DPY19–CMT2-Fab–anti-Fab Nb complex. **f**, Overview of the EM data processing and structure determination pipeline using RELION 3.1. **g**, Representative cryo-EM micrograph. **h**, Spatial distribution of particles in the final iteration of 3D refinement. **i**, Resolution estimation of final map via Fourier shell correlation, as calculated in RELION 3.1. **j**, Refined, and B-factor sharpened EM map, colored by local resolution estimation as calculated in RELION 3.1. **k**-**o**, Structure determination of acceptor peptide and Dol_25_-P-C-Man bound *Ce*DPY19–CMT2-Fab–anti-Fab Nb complex. **k**, Overview of the EM data processing and structure determination pipeline using RELION 3.1 and if indicated cryoSPARC v3.2. **l**, Representative cryo-EM micrograph. **m**, Spatial distribution of particles in the final iteration of 3D refinement. **n**, Resolution estimation of final map via Fourier shell correlation, as calculated in cryoSPARC v3.2. **o**, Resolution estimation of final map via Fourier shell correlation, as calculated in RELION 3.1. Source data

To reveal how CMTs select and bind acceptor sequons, we determined a cryo-EM structure of *Ce*DPY19 bound to a synthetic octapeptide (WEHI‐1886493, **7**), which contains a WxxW sequon: Trp(0)-Ala(+1)-Lys(+2)-Trp(+3). The numbers in parentheses refer to the location relative to the tryptophan Trp(0) that is modified by *Ce*DPY19. A fluorescently labeled version of this peptide (WEHI-1886494) was found to be a suitable substrate of *Ce*DPY19 in vitro (Extended Data Fig. 1b). The resolution of the acceptor peptide-bound complex was 2.7 Å (Fig. 3, Extended Data Fig. 5 and Supplementary Table 1), and the peptide was well-resolved, providing complete coverage of the WxxW sequon (Fig. 3a). To test the functional relevance of the enzyme–peptide interactions observed in our structure, we determined the activity of selected *Ce*DPY19 mutants using a semiquantitative, yeast-based cellular assay^26^. Each *Ce*DPY19 mutant was coexpressed with RNase2, a substrate of CMTs that can be used as a reporter of *C*-mannosylation activity, within *Pichia pastoris*, an organism that is naturally devoid of *C*-mannosylation activity. The RNase2 reporter protein was affinity-purified from culture supernatants and the occupancy of its single *C*-mannosylation site determined by MS after trypsin digestion of the samples (Fig. 4).

The acceptor peptide binds into a groove between the endoplasmic reticulum–luminal loops and the luminal dome of *Ce*DPY19. While grooves for binding peptides are present at similar general locations in OST and PMT1/2 (refs. 28,29,32), the shape and the interactions with the acceptor peptide are distinct in *Ce*DPY19, providing selectivity for the WxxW sequon. The shape of the groove forces the main chain of the acceptor peptide to bend sharply next to Trp(0), which is incompatible with any secondary protein structure. This observation rationalizes why CMTs exclusively process unfolded proteins^3,23^, since neither an α-helix nor a β-strand would fit into the binding pocket of the enzyme; yet, *C*-mannosylation is often found thrombospondin type 1 repeats that have an extended polypeptide backbone conformation that closely resembles beta strands^4^.

The backbone of the acceptor peptide forms several H-bonds to side chains of *Ce*DPY19. The indole moiety of Trp(+3) is wedged into a deep cavity and held in place by cation–π interactions with the flanking residues Arg211 and Lys473 (Fig. 3a inset). Mutating either of those residues to alanine led to a near-complete (90–100%) drop in protein *C*-mannosylation (Fig. 4). Notably, a mutation of the equivalent arginine (Arg290) to histidine in the human *Hs*DPY19L2 paralog represents the most frequently reported missense mutation in globozoospermic patients^41^. The side chain of Ala(+1) of the acceptor peptide points into a shallow cavity of the enzyme, where larger side chains would clash and therefore interfere with sequon binding. This rationalizes the observed preference for smaller side chains at position (+1) of the sequon^3^. In contrast, the side chain of Lys(+2) points into the solvent, which explains the high tolerance of CMTs to sequence variability reported for position (+2) of the WxxW sequon^3^. The indole group of the acceptor tryptophan, Trp(0), fits snugly into a groove formed mainly by the aromatic side chains Tyr395, Phe401 and Tyr578, forming a network of π–π stacking interactions. The backbone carbonyl of Pro576 forms an H-bond to N1 of the indole of Trp(0), providing a key contact to the substrate.

To rationalize the acceptor sequon variability at position (+3) of the four human CMT paralogs, we compared our peptide-bound *Ce*DPY19 structure to the models of *Hs*DPY19L1-L4, as predicted by AlphaFold^42^ (Fig. 3b). The predicted peptide-binding sites of *Hs*DPY19L1 and *Hs*DPY19L2 are similar to those of *Ce*DPY19, which is in line with the finding that *Hs*DPY19L1 recognizes WxxW sequons^5^. While the substrate specificity of *Hs*DPY19L2 has not been experimentally demonstrated, its similarity to *Ce*DPY19 suggests that *Hs*DPY19L2 is an active testis-specific CMT that preferably glycosylates the WxxW sequon. In contrast, *Hs*DPY19L3 recognizes the sequon WxxC, which contains a cysteine at the (+3) position^5^. In the *Ce*DPY19 structure, a leucine residue (Leu474) is present near Trp(+3) of the bound peptide, forming the ‘floor’ of the Trp(+3) indole-binding pocket. The equivalent residue in *Hs*DPY19L3 is a tyrosine (Tyr485), which would clash with the indole moiety of Trp(+3) (Fig. 3b). This likely explains why *Hs*DPY19L3 cannot process the WxxW sequon^5^. Instead, it is plausible that the hydroxyl group of Tyr485 of *Hs*DPY19L3 H-bonds to Cys(+3) in the WxxC sequon. Finally, *Hs*DPY19L4 has the least similar active site compared to *Ce*DPY19 (Fig. 3b). Like *Hs*DPY19L3, the predicted model of *Hs*DPY19L4 features a tyrosine residue (Tyr488 in *Hs*DPY19L4) at the position of Leu474 in *Ce*DPY19. Although this does not reveal the substrate specificity of *Hs*DPY19L4, it suggests that *Hs*DPY19L4 is unlikely to recognize and process the canonical WxxW sequon, and is more likely to have a preference for amino acids with smaller side chains at the (+3) position. We conclude that *Ce*DPY19-Leu474 and its equivalent residues in CMT paralogs are the key determinant of CMT acceptor sequon preference at the (+3) position.

### Active site structure and mechanism of Dol-P-Man recognition

Several side chains in the vicinity of the acceptor indole are likely involved in catalysis. Glu71 is well-positioned to abstract a proton from the C2 of the Trp(0) indole group at some point during the glycosylation reaction: no other nearby residue could act as general base during catalysis. Mutating Glu71 to Ala, Gln, Leu or Met abolished *Ce*DPY19 activity (Fig. 4), providing strong support for an essential role of Glu71 in catalysis: most likely as a catalytic base. Mutating Glu71 to Asp only resulted in a 20% drop in protein *C*-mannosylation. Notably, the human paralog *Hs*DPY19L4 contains an aspartate (Asp95) at this position rather than a glutamate, revealing that this protein is likely a competent enzyme and that nature has explored and adopted both residues as catalytic bases for *C*-mannosylation (Fig. 3b and Extended Data Fig. 4). As expected from our in vitro assay, and unlike in OST^32^ or PMT1/2 (ref. 29), no obvious residues for coordinating metal ions were found in the *Ce*DPY19 active site.

**Fig. 5:**
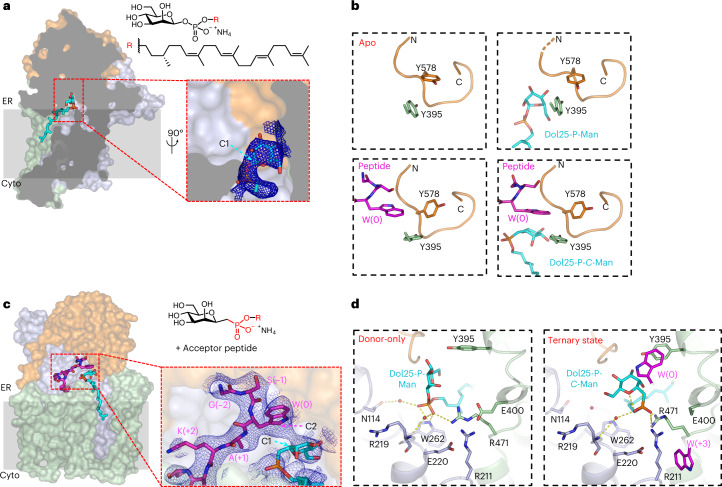
A trapped ternary complex explains donor-substrate recruitment and activation. **a**, Surface representation of Dol25-P-Man-bound *Ce*DPY19 structure colored as in Fig. 3a, with Dol25-P-Man (chemical structure on the right, top) in stick representation (cyan). The front of *Ce*DPY19 was clipped for clarity. The inset shows the phosphate and mannose moieties of Dol25-P-Man, and the blue mesh depicts the EM density map. The anomeric C1 of the mannose moiety is indicated by an arrow. **b**, Backbone structure of loop LDLβ2-α3 (His575-Arg584, colored orange) in the four *Ce*DPY19 structures. Bound substrates are shown in sticks and labeled. Residue Tyr395 likely acting as a peptide sensor is shown in sticks and labeled. **c**, Surface representation of the ternary complex structure containing the phosphonate analog Dol25-P-*C*-Man (chemical structure on the right, top) and acceptor peptide, both shown as sticks. The inset shows bound ligands, with the EM density map shown as a blue mesh. **d**, Reorientation of the donor substrate in response to peptide binding to the enzyme. Left shows the Dol25-P-Man-bound *Ce*DPY19 structure. Right shows the ternary complex structure. Red spheres indicate ordered water molecules. Selected side chains, as well as the substrates, are shown as sticks. Bound ligands are shown as sticks and labeled. Dashed yellow lines indicate distances from 2.5 to 3.8 Å.

**Extended Data Fig. 6:**
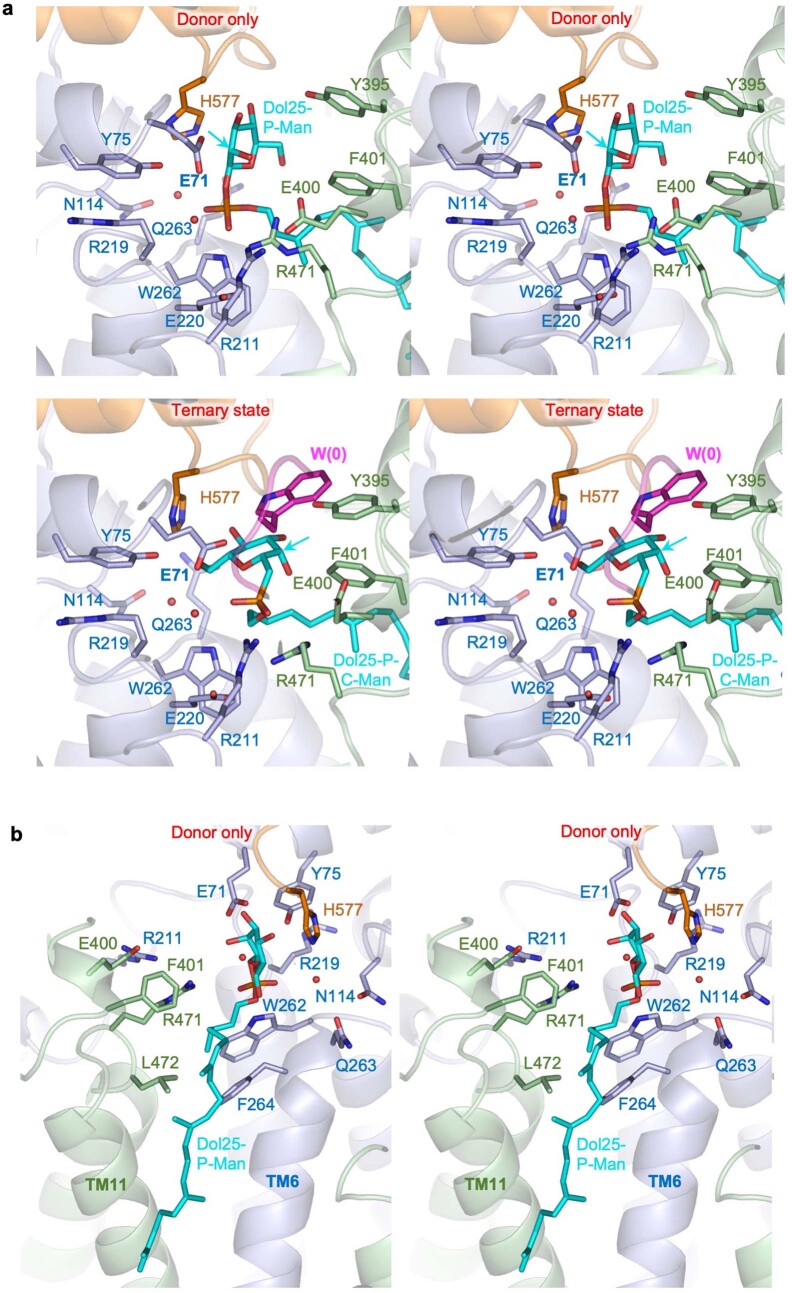
Donor substrate recruitment by CMT *Ce*DPY19. *Ce*DPY19 and its substrates are colored as in Fig. 5 and depicted in wall-eyed stereo representation. *Ce*DPY19 is shown in ribbon representation. The substrates and selected close residues are shown in stick representation. Arrows depict the C2 carbon of the mannose moiety. **a**, Mannose recognition in the structures of Dol_25_-P-Man-bound *Ce*DPY19 (top) and Dol_25_-P-C-Man- plus acceptor peptide-bound *Ce*DPY19 (bottom). **b**, Dolichyl recognition in the structure of Dol_25_-P-Man-bound *Ce*DPY19.

To understand how CMTs recognize their donor substrate, we determined a 3.0 Å resolution structure of *Ce*DPY19 bound to the synthetic, water-soluble donor-substrate analog Dol25-P-Man (ref. 33) (Fig. 5a, Extended Data Fig. 5 and Supplementary Table 1). The substrate is recruited to the active site via a tunnel formed by EL5. At the active site, the mannose moiety is partially solvent-exposed but also forms several hydrogen bonds with the enzyme. The selectivity for Dol-P-Man over Dol-P-Glc appears to be ensured by the presumed catalytic base residue Glu71, the side chain of which would clash with the equatorial C2 hydroxyl of the glucose moiety if Dol-P-Glc was bound instead of Dol-P-Man (Extended Data Fig. 6a).

The phosphate moiety, which is the leaving group of Dol-P-Man, is coordinated by a salt bridge to Arg471 and by an H-bond to the indole NH of Trp262 (Extended Data Fig. 6a). We found that the mutations R471A and R471Q abolished *Ce*DPY19 activity (Fig. 4), demonstrating an important role for Arg471 in recruiting the donor substrate and/or catalysis. The dolichyl moiety of bound Dol25-P-Man fits into a hydrophobic groove formed by TM6 and TM11 that is lined with the side chains of the conserved hydrophobic residues Trp262, Phe264, Leu472 and Phe401 from EL5 (Extended Data Fig. 6b). The mutations F264A and F401A led to a >50% reduction in protein *C*-mannosylation (Fig. 4), suggesting that recruitment of Dol-P-Man involves the specific recognition, binding and partial extraction of the dolichyl moiety from the membrane.

### Trapped ternary complex reveals donor recruitment and activation

A comparison of the independently determined structures of peptide-bound and Dol25-P-Man-bound *Ce*DPY19 revealed that the acceptor tryptophan of the bound peptide would clash with the mannose moiety of Dol25-P-Man (Fig. 5b). This suggests that conformational changes are required in the active site for the two substrates to bind simultaneously and for catalysis to proceed. To visualize these changes, we trapped the enzyme in a ternary complex using a nonhydrolyzable and thus nonreactive donor substrate analog containing a phosphonate group, termed Dol25-P-*C*-Man **8** (Fig. 5c). This prevents turnover, and the resulting ternary complex corresponds to a pseudo-Michaelis complex.

**Fig. 6:**
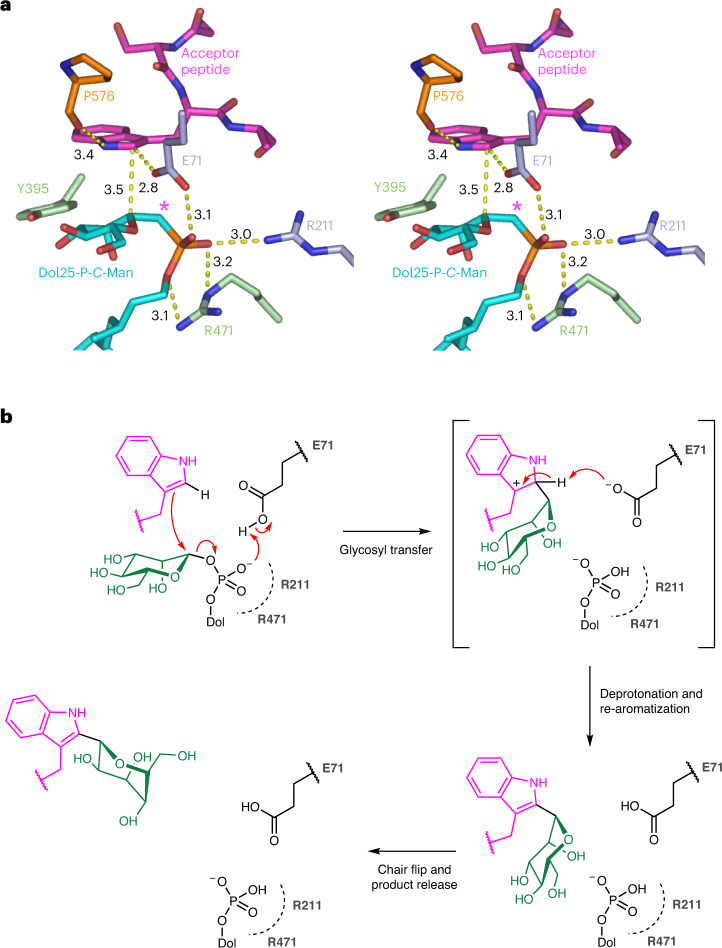
Catalytic mechanism. **a**, Stereo view of *Ce*DPY19 ternary complex, with bound peptide colored magenta, bound Dol25-P-*C*-Man colored in cyan and key residues shown as sticks and labeled. An asterisk depicts the carbon atom of the phosphonate analog Dol25-P-*C*-Man. Dashed yellow lines indicate distances between key atoms in Å. **b**, Schematic of the proposed catalytic mechanism for CMT. See text for explanation.

The structure, determined at 3.6 Å, revealed an active site conformation that was more similar to acceptor peptide-bound than to donor-substrate-bound *Ce*DPY19 (Fig. 5b). The orientation of the bound peptide and its interactions with the enzyme were very similar to the peptide-bound *Ce*DPY19 structure (Fig. 5b, Extended Data Fig. 5 and Supplementary Table 1). In contrast, key conformational changes were observed for bound Dol25-P-*C*-Man compared to Dol25-P-Man-bound *Ce*DPY19. First, the phosphonate moiety is shifted by roughly 4 Å toward the two arginines Arg471 and Arg211 and the catalytic Glu71 (Extended Data Fig. 6). Second, the mannose moiety adopts a ‘bent-back’ conformation (Fig. 5d) that allows for the simultaneous binding of the donor substrate and the acceptor peptide. As a result, the anomeric C1 carbon of the mannose moiety is at a distance of roughly 3.5 Å from the C2 carbon of the acceptor tryptophan and optimally positioned for an electrophilic attack (Fig. 6a and Extended Data Fig. 6a).

The bent-back conformation of Dol25-P-*C*-Man appears to be stabilized by the active site loop LDLβ2-α3. Because this loop strongly interacts with bound peptide, the activation of the donor substrate appears allosterically induced by acceptor peptide binding (Fig. 5c). Similar bent-back conformations have been observed in nucleoside-diphosphate-linked hexose donors bound to enzymes of the glycosyltransferase superfamily B (GT-B), including PimA^43^, MshA^44^, HepE^45^ and PglH^46^. Our findings reveal how the donor substrate only adopts an active conformation on acceptor peptide binding, providing a mechanism to prevent the futile hydrolysis of Dol-P-Man in the absence of an acceptor peptide. No other GT-C structure has been reported in both the donor-only and the ternary complex state. The only other example of a donor-only bound GT-C, ALG6 bound to Dol-P-Glc, was also observed in a catalytically inactive state^33^. Given the structural similarity of donor-substrate binding sites in GT-Cs^33^ and a proposed common mode of donor recruitment^47^, we postulate that inactive donor resting states and acceptor-mediated donor activation is conserved in GT-C enzymes.

### Catalytic mechanism

Our structural and functional findings provide sufficient molecular detail to propose a mechanism for CMT-catalyzed tryptophan *C*-mannosylation (Fig. 6b). The reaction catalyzed by tryptophan CMT can be considered an electrophilic aromatic substitution at C2 of the indole, with inversion of configuration at the anomeric (C1) carbon of the mannose moiety^22^. The enzyme ensures regioselectivity by placing C1 of the mannose donor in close proximity to C2 of the indole acceptor. As the basicity of the carboxylate side chain of the catalytic Glu71 is likely insufficient for direct deprotonation of the indole at C2, we propose that this occurs after the mannosyl transfer step. The reaction can be thought to occur in three steps. In step 1, the enzyme activates the mannosyl donor by stabilizing the negative charge of the departing dolichylphosphate leaving group via interactions with Arg211 and Arg471, and potentially through protonation by Glu71. This facilitates an attack of the indole on the anomeric carbon of mannose, forming a *C*-glycosidic bond with inversion of anomeric configuration. The resulting cationic intermediate is resonance-stabilized. In step 2, the C2 proton of the cationic intermediate can now be plausibly abstracted by Glu71 to rearomatize the indole and generate the reaction product, with Glu71 completing its role as general acid–base in the reaction. While it is not clear to what extent steps 1 and 2 are concerted, mechanistic studies of other glycosyltransferases suggest that late transition states with substantial ionic character are not uncommon during glycosyl transfer^48^. The presumed resonance-stabilized cationic intermediate may be further stabilized by H-bonding interactions between the indole N1 and the backbone carbonyl of Pro576 and via a π-stacking network with Tyr395, Phe401 and Tyr578. In step 3, the observed geometric arrangements of the substrates and steric restrictions of the active site suggest an immediate reaction product with the mannose in a ^4^C_1_ conformation. Given that the energetically preferred conformation of the *C*-linked mannose is ^1^C_4_ rather than ^4^C_1_ (ref. 49), product release from the enzyme might coincide with a conformational flip of the mannose to ^1^C_4_. This minimizes the potential for product inhibition.

Mechanistically, CMT and GT-C members such as OST and PMT1/2 share similar spatial arrangements of donor and acceptor substrate, as well as analogous carboxylic acid residues (Glu71 in *Ce*DPY19) as a general base. They all promote glycosyl transfer by stabilizing (pyro)phosphate leaving groups through interactions with cationic residues and metals. Unlike OST and PMT1, CMT appears to have evolved metal independence by repurposing its catalytic carboxylic acid residue as a general acid–base that can promote phosphate departure through protonation then deprotonate the glycosylated intermediate to generate product. However, the most significant difference between CMT and other GT-C members, such as OST and PMT1/2, is that the latter catalyze nucleophilic displacements, while the former catalyzes an electrophilic aromatic substitution. Thus, while OST has evolved unique mechanisms to enhance amide nucleophilicity^32^, CMT has evolved the means to direct electrophilic attack at the C2 position of indole, stabilize the cationic intermediate, and regenerate indole aromaticity via deprotonation. This mechanism of enzyme-mediated carbon–carbon bond formation on ribosomal peptides is unique, with the closest related example being the prenylation of tryptophan-derived secondary metabolites via electrophilic aromatic substitution by fungal dimethylallyltryptophan synthases^50^. However, the fold, chemistry, substrate preferences and mechanisms of the dimethylallyltryptophan synthases are entirely different from those of the CMTs.

## Discussion

The *Ce*DPY19 structures presented here define the architecture of tryptophan CMTs, provide the structural basis of acceptor sequon binding in nascent polypeptide chains and of mannose donor-substrate recognition, and reveal how the donor substrate is activated by the enzyme. These data enabled us to propose a reaction mechanism for *C*-glycosidic bond formation, thereby addressing the most substantial gap in our understanding of protein glycosylation chemistries. The structures also provide a framework for understanding the recruitment and activation of lipid-linked carbohydrate donors by GT-C superfamily enzymes. Our findings rationalize the protein substrate preferences of the human CMT paralogs *Hs*DPY19L1/L3, provide clues as to the preferences of *Hs*DPY19L2/L4 and help understand mutations that cause CMT-deficiency-associated male infertility. Finally, our substrate-bound structures provide a strong foundation for the development of CMT inhibitors. Such molecules would be invaluable for exploring the biology of tryptophan *C*-mannosylation through paralog-specific inhibition of CMT enzymes to better understand the physiological role of tryptophan *C*-mannosylation in cell–cell communication and tissue development. They may also have applications as anti-parasite drugs (for example, against toxoplasmosis, malaria or helminth infections) or as male contraceptives through the specific inhibition of human *Hs*DPY19L2.

## Methods

### Cell culture

Sf9 cells were cultured in serum-free SF4 medium. *GS115 P. pastoris* cells were cultured in yeast extract peptone dextrose (YPD)-medium. M13-KO7 Phage were cultured in *Escherichia coli* XL1-Blue cells, in 2xYT medium.

### Overexpression and purification of *Ce*DPY19

A synthetic gene construct encoding *C. elegans* DPY19 (*Ce*DPY19) with a C-terminal FLAG_3_ tag^26^ was cloned into a pOET1 vector (Oxford Expression Technologies) and was expressed in *Spodoptera frugiperda* (Sf9) cells transfected with baculovirus that was generated using flashBAC GOLD (Oxford Expression Technologies). Cells were cultured in serum-free SF4 medium at 27 °C. Cells were transfected at a density of 1 × 10^6^ cells per ml and were harvested after 3 days. For purification, the cells were resuspended in 50 mM HEPES pH 7.4, 150 mM NaCl with 0.1 mg ml^−1^ DNAseI, 1:100 protease inhibitor cocktail (Sigma), 0.1 mg ml^−1^ PMSF and were lysed by dounce homogenization before solubilization by addition of 0.5% lauryl maltose neopentyl glycol (LMNG, Anatrace) 0.05% cholesteryl hemisuccinate (CHS, Anatrace) and 10% glycerol. After 1 h of solubilization, cell debris were pelleted by centrifugation at 100,000*g* in a type T45 Ti rotor (Beckmann). The supernatant was added to ANTI-FLAG M2-Affinity Gel (SigmaAlrich) and was incubated for 1 h. The affinity gel then was washed with 2 × 20 column volumes of washing buffer (40 mM HEPES pH 7.4, 150 mM NaCl, 0.01% LMNG, 0.001% CHS). Then the protein was eluted by incubation with washing buffer, supplemented with 0.3 mg ml^−1^ FLAG-peptide for 1 h. The protein was further purified using size-exclusion chromatography (SEC) and thereby desalted into 20 mM HEPES pH 7.4, 150 mM NaCl, 0.01% LMNG, 0.001% CHS.

### In vitro glycosyl transfer assays for *Ce*DPY19

Reactions of *Ce*DPY19 were carried out in reaction buffer (40 mM HEPES pH 7.4, 150 mM NaCl, 0.01% LMNG and 0.001% CHS). Purified *Ce*DPY19 (50 nM) was mixed with 50 mM Dol25-P-Man, 10 mM peptide and optionally 5 mM of MnCl_2_, MgCl_2_ or EDTA. For reactions in the presence of EDTA, *Ce*DPY19 was preincubated for 1 h on ice with the chelator before adding donor and acceptor substrates. The reactions were incubated for a total time of 42 h at 20 °C. Subsequently, the reactions were stopped by 50-fold dilution in Laemmli buffer and were subjected to Tricine–SDS–PAGE^51^.

### Enzymatic biotinylation of *Ce*DPY19

*Ce*DPY19 was fused to a C-terminal Avitag and purified as described above. Then *Ce*DPY19 was biotinylated in biotinylation buffer (20 mM HEPES pH 7.4, 150 mM NaCl, 50 mM bicine pH 8.3, 10 mM Mg acetate, 10 mM ATP, 0.25 mM biotin 0.01% w/v LMNG, 0.001% w/v CHS) with 2 µM BirA protein, overnight at 4 °C. Subsequently, SEC was used to exchange the buffer to 20 mM HEPES pH 7.4, 150 mM NaCl, 0.01% LMNG, 0.001% CHS and to remove excess BirA, biotin and ATP. The biotinylated protein was flash frozen and stored at −80 °C.

### Phage display selection

Biotinylated *Ce*DPY19 was used for the phage display selection. Pull-down experiments on Streptavidin MagneSphere paramagnetic particles (Promega) showed quantitative biotinylation of the target used in selection. Phage display selection was performed at 4 °C according to published protocols^52^. The selection buffer was 20 mM HEPES pH 7.4, 150 mM NaCl, 0.01% LMNG, 0.001% CHS and 0.5% BSA. In the first round, 250 nM of the target was immobilized on 250 µl of magnetic beads. Then, 100 μl of a phage library E^35^ containing 10^12^–10^13^ virions were added to the Streptavidin beads and incubated for 30 min. The resuspended beads containing bound phages were washed extensively and then used to infect log-phase *E. coli* XL1-Blue cells. Phages were amplified overnight in 2xYT media with 50 µg ml^−1^ ampicillin and 10^9^ pfu ml^−1^ of M13-KO7 helper phage. To obtain binders of high affinity and specificity, three additional rounds of selection were performed with decreasing the target concentration in each round (second round 125 nM, third round 62.5 nM and fourth round 12.5 and 6.5 nM) using the amplified pool of phages of the preceding round as the input. Selection from second to fourth rounds was done on a KingFisher Purification System (Thermo Scientific) using a solution capture method where the target was premixed with the amplified phage pool and then Streptavidin beads were added to the mixture. From the second round onward, the bound phages were eluted using 100 mM glycine, pH 2.7. This harsh elution technique often results in the elution of nonspecific and Streptavidin binders. To eliminate them, the precipitated phage pool from the second round onward were negatively selected against 100 µl of Streptavidin beads before adding to the target. The precleared phage pool was then used as an input for the selection.

### Single-point enzyme-linked immunosorbent assay (ELISA)

ELISA experiments were performed at 4 °C in 96-well plates coated with 50 µl of 2 µg ml^−1^ neutravidin in Na_2_CO_3_ buffer, pH 9.6 and subsequently blocked by 0.5% BSA in PBS. A single-point phage ELISA was used to rapidly screen the binding of the obtained Fab fragments in phage format. Colonies of *E. coli* XL1-Blue harboring phagemids from the fourth round of selection were inoculated directly into 500 μl of 2xYT broth supplemented with 100 μg ml^−1^ ampicillin and M13-KO7 helper phage. The cultures were grown overnight at 37 °C in a 96-deep-well block plate. The ELISA buffer was identical to that used in selection. The experimental wells in the ELISA plates were incubated with 30 nM *Ce*DPY19 in ELISA buffer for 15 min. Only buffer was added to the control wells. Overnight culture supernatants containing Fab phage were diluted tenfold in ELISA buffer. The diluted phage supernatants were then transferred to ELISA plates that were preincubated with biotinylated target and washed with ELISA buffer. The ELISA plates were incubated with the phage for another 15 min and then washed with ELISA buffer. The washed ELISA plates were incubated with a 1:1 mixture of mouse anti-M13 monoclonal antibody (catalog no. 27-9420-01, GE Healthcare, 1:5,000 dilution in ELISA buffer) and peroxidase-conjugated goat antimouse IgG (catalog no. 115-035-003, Jackson Immunoresearch, 1:5,000 dilution in ELISA buffer) for 30 min. The plates were again washed, developed with 3,3',5,5'-tetramethylbenzidine substrate and then quenched with 1.0 M HCl, and the absorbance at 450 nm was determined. The background binding of the phage was monitored by the absorbance from the control wells.

### Sequencing, cloning, expression and purification of Fab fragments

From phage ELISA, clones (selected based on a high ratio of ELISA signal of target binding to background) were sequenced at the DNA Sequencing Facility at the University of Chicago. More than 80 unique clones were obtained. Twelve unique clones with the highest ELISA signal and least background were subcloned in pRH2.2, an isopropyl-β-d-thiogalactoside inducible vector for expression of Fabs in *E. coli. E. coli* C43 (Pro+) cells^36^ were transformed with sequence-verified clones of Fab fragments in pRH2.2. Fab fragments were grown in Terrific Broth autoinduction media with 100 μg ml^−1^ ampicillin overnight at 30 °C. Collected cells were kept frozen at −80 °C until use. Frozen pellets were resuspended in PBS supplemented with 1 mM PMSF and 1 μg ml^−1^ DNaseI. The suspension was lysed by ultrasonication. The cell lysate was incubated at 65 °C for 30 min followed by centrifugation. The supernatant was filtered through a 0.22 µm filter and loaded onto a HiTrap Protein L 5-ml column preequilibrated with lysis buffer (20 mM HEPES buffer, pH 7.5, 500 mM NaCl). The column was washed with 10 column volumes of lysis buffer followed by elution of Fab fragments with elution buffer (100 mM acetic acid). Fractions containing protein were directly loaded onto a Resource S 1-ml column preequilibrated with buffer A (50 mM sodium acetate, pH 5.0) followed by washing with 10 column volumes with buffer A. Fab fragments were eluted with a linear gradient 0–50% of buffer B (50 mM sodium acetate, pH 5.0, 2.0 M NaCl). Affinity and ion-exchange chromatography were performed using an automated program on a ÄKTA explorer system. Purified Fabs were dialyzed overnight against 20 mM HEPES, pH 7.4, 150 mM NaCl. The quality of purified Fab fragments was analyzed by SDS–PAGE.

### Multipoint protein ELISA for half-maximum effective concentration (EC_50_) determination

Multipoint ELISA was performed at 4 °C to estimate the affinity of the Fabs to *Ce*DPY19. 20 mM HEPES pH 7.4, 150 mM NaCl, 0.01% LMNG, 0.001% CHS supplemented with 0.5% BSA was used as the ELISA buffer. Then 30 nM of target immobilized on a neutravidin coated ELISA plate was incubated with threefold serial dilutions of the purified Fabs starting from 4 μM for 20 min. The plates were washed, and the bound *Ce*DPY19-Fab complexes were incubated with a secondary HRP-conjugated Pierce recombinant protein L (catalog no. 32420, Thermofisher, 1:5,000 dilution in ELISA buffer) for 30 min. The plates were again washed, developed with 3,3',5,5'-tetramethylbenzidine substrate and quenched with 1.0 M HCl, and absorbance (A_450_) was determined. To determine the affinities, the data were fitted in a dose-response sigmoidal function in GraphPad Prism and EC_50_ values were calculated.

### Thermostability assays

Thermostability analysis experiments were performed as described previously^53^, with purified *Ce*DPY19 in LMNG:CHS supplemented buffer, preincubated with or without a 1.5-fold molar excess of Fab for 2 h on ice. Then the samples were incubated for 10 min at different temperatures in a PCR machine and analyzed by SEC, measuring A_280_ instead of fluorescence during SEC to assess the area under the curve of the SEC peaks of the respective samples. While a full curve was measured to determine the melting temperature (*T*_m_) of *Ce*DPY19 (35.68 °C), only two data points were measured for *Ce*DPY19-Fab complexes: one at 4 °C and one at 36 °C. Then the percentage of peak-high retention between the samples with and without Fab was compared, which allowed for a qualitative assessment of thermostabilizing effects.

### EM sample preparation

For the apo structure, purified *Ce*DPY19 was mixed with excess CMT2-Fab and excess anti-Fab nanobody^37^. After incubation overnight at 4 °C, excess Fab and nanobody were removed by SEC. Peak fractions were pooled and the *Ce*DPY19–CMT2-Fab–anti-Fab–Nb complex was concentrated to 6.5 mg ml^−1^ and used for cryo-EM grid preparation.

For the acceptor peptide-bound structure and for the Dol25-P-Man-bound structure, *Ce*DPY19–CMT2-Fab–anti-Fab–Nb complex was concentrated to 5 mg ml^−1^ (35 µM). Subsequently, either 1 mM acceptor peptide WEHI‐1886493 (Ac-Pra-GSWAKWS-NH2) or 500 µM synthetic Dol25-P-Man (ref. 33) (final concentrations) were added, and the samples were incubated for 1–2 h on ice and then used for grid preparation.

For the structure of the ternary complex *Ce*DPY19–CMT2-Fab–anti-Fab–Nb complex was concentrated to 4.9 mg ml^−1^ (34 µM) and was mixed with 500 µM Dol25-P-*C*-Man and 1 mM Ac-Pra-GSWAKWS-NH2 (final concentrations). The samples were incubated for 1 h on ice and subsequently used for grid preparation.

### EM grid preparation

Quantifoil holey carbon grids, Cu, R 1.2/1.3, 300 mesh, were glow discharged for 45 s, 25 mA using a PELCO easiGLOW glow discharger. Sample (2.5 µl) was applied to the cryo-EM grids and blotted for 1–3.5 s before plunge freezing in a liquid ethane–propane mixture with a Vitrobot Mark IV (Thermo Fisher Scientific) operated at 4 °C and 100% humidity.

### EM data collection

Data were recorded on a Titan Krios electron microscope (Thermo Fischer Scientific, second generation) operated at 300 kV, equipped with a Gatan BioQuantum 1967 filter with a slit width of 20 eV and a Gatan K3 camera. Videos were collected semiautomatically using EPU 2 software (Thermo Fisher Scientific) at a nominal magnification of ×130,000 and a pixel size 0.33 Å per pixel, in super-resolution mode. The defocus range was −0.6 to −2.8 µm. Each video contained 40 images per stack with a dose per frame of 1.21 e^−^/Å^2^.

### EM data processing, model building and refinement

For the apo structure of *Ce*DPY19, 11,725 movies were collected, corrected for beam-induced motion using MotionCor2 (ref. 54) and subjected to further processing in RELION v.3.1 (https://relion.readthedocs.io/en/latest/Installation.html). The contrast transfer function (CTF) was estimated using Gctf^55^. Using LOG-based particle picking, 7,357,737 particles were auto-picked, extracted with threefold binning (1.98 Å per pixel) and were sorted by two-(2D) and three-dimensional (3D) classification. A total of 473,614 particles were re-extracted to 0.66 Å per pixel and were subjected to another round of 3D classification. Therefrom, 384,830 particles were selected and subjected to further refinement where the Fab and the detergent micelle were masked out. Subsequent particle polishing and per-particle CTF refinement allowed refinement of the particles to 2.75 Å resolution, by masking out the Fab–Nb complex and the detergent micelle.

For the acceptor peptide-bound structure of *Ce*DPY19, 13,041 movies were collected, corrected for beam-induced motion using MotionCor2 (ref. 54) and subjected to further processing in RELION v.3.1. The CTF was estimated using Gctf^55^. Next, 9,885 micrographs were selected for further processing as they had an estimated resolution higher than 3.5 Å. Using LOG-based particle picking, 3,876,382 particles were auto-picked, extracted with threefold binning (1.98 Å per pixel) and were sorted by 2D and 3D classification. A total of 324,852 particles were re-extracted to 0.66 Å per pixel and subjected to further refinement where the Fab and the detergent micelle were masked out. Subsequent particle polishing and per-particle CTF refinement allowed to refine the particles to 2.83 Å resolution. To improve the resolution of the substrate the particles were subjected to an additional round of 3D classification and 287,795 particles were selected. Subsequent particle polishing and per-particle CTF refinement allowed to refine the particles to 2.72 Å resolution, by masking out the Fab–Nb complex and the detergent micelle.

For the Dol25-P-Man-bound structure of *Ce*DPY19, 7,874 movies were collected, corrected for beam-induced motion using MotionCor2 (ref. 54) and subjected to further processing in RELION v.3.1. The CTF was estimated using Gctf^55^. Then 7,115 micrographs were selected for further processing as they had an estimated resolution higher than 3.5 Å. Using LOG-based particle picking, 2,543,900 particles were auto-picked, extracted with threefold binning (1.98 Å per pixel) and were sorted by 2D and 3D classification. A total of 301,020 particles were re-extracted to 0.66 Å per pixel and subjected to further refinement where the Fab and the detergent micelle were masked out. Subsequent particle polishing and per-particle CTF refinement allowed to refine the particles to 2.97 Å resolution. To improve the resolution of the substrate the particles were subjected to an additional round of nonuniform refinement in cryoSPARC v.3.2 (https://cryosparc.com/) with optimized per-particle defocus and optimized per-group CTF parameters yielding a final resolution of 2.99 Å, using a soft mask around the entire particle.

For the structure of the ternary complex of *Ce*DPY19, 21,730 movies were collected, corrected for beam-induced motion using MotionCor2 (ref. 54) and subjected to further processing in RELION v.3.1. The CTF was estimated using Gctf^55^. Next, 12,385 micrographs were selected for further processing as they had an estimated resolution higher than 3.5 Å. Using LOG-based particle picking, 4,309,649 particles were auto-picked, extracted with threefold binning (1.98 Å per pixel) and were sorted by 2D and 3D classification. A total of 276,165 particles were re-extracted to 0.66 Å per pixel and subjected to further refinement where the Fab and the detergent micelle were masked out. Subsequent particle polishing and per-particle CTF refinement allowed refinement of the particles to 3.2 Å resolution when masking out the Fab–Nb complex and the detergent micelle. To improve the resolution of the substrates the particles were subjected to an additional round of nonuniform refinement in cryoSPARC v.3.2 (https://cryosparc.com/) with optimized per-particle defocus and optimized per-group CTF parameters yielding a final resolution of 3.31 Å. To identify particles with a high occupancy of Dol25-P-*C*-Man, the particles were subjected to 3D variability in cryoSPARC v.3.2 analysis while masking out the Fab and the detergent micelle. Next, 57,289 particles were selected therefrom and were subjected to nonuniform refinement in cryoSPARC v.3.2 with optimized per-particle defocus and optimized per-group CTF parameters yielding a final overall resolution of 3.63 Å, using a soft mask around the entire particle. Local resolution estimates for all structures were calculated in RELION 3.1.

Atomic coordinates were built manually in Coot (https://www.ucl.ac.uk/~rmhasek/coot.html), were refined in PHENIX (http://www.phenix-online.org/), and were validated using MolProbity (http://molprobity.biochem.duke.edu/). The model of apo *Ce*DPY19 was built de novo and the ligand-bound *Ce*DPY19 models were built based on the coordinates of the apo structure. The structure of the CMT2-Fab–anti-Fab–Nb complex was built based on a published model of a Fab–anti-Fab–Nb complex^37^. Ligands were generated using eLBOW (https://phenix-online.org/documentation/reference/elbow.html).

### Solid phase peptide synthesis

Solid phase peptide synthesis was performed on a CEM Liberty Blue Automated Microwave Peptide Synthesizer. Rink amide resin (0.68 mmol g^−1^ loading) was swollen in N, N-dimethylformamide (DMF) for 1 h then washed with DMF (10 ml) and CH_2_Cl_2_ (2 × 10 ml) before coupling of the first Fmoc-protected amino acid. Unless otherwise stated, Fmoc-protected amino acids were coupled under the following condition: 4 eq. Fmoc-Xxx-OH, 1.25 eq. DIPEA, 5 eq. DIC, 5 eq. Oxyma, microwave 90 °C, 4 min. Fmoc deprotection was accomplished by treating the resin with 20% (v/v) pyrrolidine in DMF at 75 °C for 5 min, then washing three times with DMF. N-terminal capping acetyl was achieved by treating the resin with Ac_2_O/DIPEA/DMF (5/5/90, v/v/v, 5 ml) at 22 °C for 30 min. Cleavage of the completed peptide from the resin was achieved with a solution of TFA/*i*Pr_3_SiH/H_2_O (95/2.5/2.5, v/v/v, 5 ml) at 40 °C for 40 min. The cleavage solution was concentrated to 2 ml and the product precipitated by the addition of ice-cold Et_2_O (4 ml). The crude peptide precipitate was collected and purified by preparative reversed-phase high-performance liquid chromatography (HPLC) using a system comprising a Waters ZQ 3100 mass detector, 2545 pump, SFO system fluidics organizer, 2996 diode array detector and 2767 sample manager. Conditions for preparative LC–MS were as follows: the column was a Xbridge TM prep C18 OBD 5 µm 19 × 100 mm; various mobile phase gradients (solvent A 0.1% HCO_2_H in H_2_O; solvent B 0.1% HCO_2_H in MeCN); flow rate was 20 ml min^−1^ and ultraviolet-visible light (UV-vis) detection was 214 and 254 nm. Quality control data for all peptides are provided in the Supplementary Information.

### TAMRA-peptide CuAAC-mediated conjugation

The following stock solutions were prepared fresh: 100 mM peptide substrate in dimethylsulfoxide (DMSO); 100 mM 5-TAMRA azide (Click Chemistry Tools, CAS no. 1006592-61-5) in DMSO; 50 mM CuSO_4_ in H_2_O; 50 mM tris-hydroxypropyltriazolylmethylamine in H_2_O and 1 M sodium ascorbate in H_2_O. For each conjugation reaction the following were combined in a 1.5-ml microcentrifuge tube: 200 µl of peptide stock, 200 µl of 5-TAMRA azide stock, 40 µl of CuSO_4_ stock, 40 µl of tris-hydroxypropyltriazolylmethylamine stock and 200 µl of sodium ascorbate stock. The reaction was mixed well by vortex and protected from light for 30 min at 22 °C, at which point HPLC–MS revealed that the reaction was complete. This sample was purified by preparative reversed-phase HPLC using a system composed of a Waters ZQ 3100 mass detector, 2545 pump, SFO system fluidics organizer, 2996 diode array detector and 2767 sample manager. Conditions for preparative LC–MS were as follows: the column was a Xbridge TM prep C18 OBD 5 µm 19 × 100 mm; various mobile phase gradients (solvent A 0.1% HCO_2_H in H_2_O; solvent B 0.1% HCO_2_H in MeCN); flow rate 20 ml min^−1^ and UV-vis detection was 214 and 254 nm. Quality control data for all peptides are provided in the Supplementary Information.

### LC–MS analysis of glycopeptides

Here, 2 μl of samples were analyzed on a calibrated Q-Exactive mass spectrometer (Thermo Fischer Scientific) coupled to a nano-Acquity UPLC system (Waters). Peptides were resuspended in 2.5% acetonitrile with 0.1% formic acid and loaded onto an Acclaim PepMap 100 trap column (75 μm × 20 mm, 100 Å, 3 μm particle size) and separated on a nano-ACQUITY UPLC BEH130 C18 column (75 μm × 250 mm, 130 Å, 1.7 μm particle size), at a constant flow rate of 300 nl min^−1^, with a column temperature of 50 °C and a linear gradient of 2–60% acetonitrile/0.1% formic acid in 20 min, and then 60–98% acetonitrile/0.1% formic acid in 5 min, before being held isocratically for another 5 min. The mass spectrometer was operated under data-dependent acquisition, one scan cycle composed of a full-scan MS survey spectrum, followed by up to 12 sequential higher energy collisional dissociation (HCD) tandem MS (MS/MS) on the most intense signals above a threshold of 1 × 10^4^. Full-scan MS spectra (600–2,000 *m/z*) were acquired in the FT-Orbitrap at a resolution of 70,000 at 400 *m/z*, while HCD MS/MS spectra were recorded in the FT-Orbitrap at a resolution of 35,000 at 400 *m/z*. HCD was performed with a target value of 1 × 10^5^ and normalization collision energy 25 was applied. automated gain control (AGC) target values were 5 × 10^5^ for full Fourier transform-MS. For all experiments, dynamic exclusion was used with a single repeat count, 15 s repeat duration and 30 s exclusion duration. There was one clean run between samples.

### Chemical synthesis of phosphonate donor mimic Dol25-P-*C*-Man

In brief, β-d-mannosyl phosphonate **13** was synthesized from allyl 2,3,4,6-tetra-*O*-acetyl-α-d-mannopyranoside **9** in a nine-step sequence based on a previously described method^56^. Notably, installing isopropylidene protecting groups was essential to achieve a high degree of anomeric control during the key Horner–Wadsworth–Emmons phosphonate insertion reaction. The α-anomer was readily separated after cleavage of the less stable 4,6-*O*-isopropylidene protecting group (reaction schemes and compounds are shown in the Supplementary Information).

Finally, trichloroacetonitrile activation of glycosyl donor **13**, coupling with (*S*)-farnesylcitronellol^57^ **14** and global deprotection as previously reported^33^ allowed the isolation of Man-CP-C25-farnesylcitronellyl **8** (Supplementary Information). Details of the synthesis are provided in the Supplementary Information.

### Cloning *Ce*DPY19 mutants

Site-directed mutagenesis of *Ce*DPY19 was accomplished by PCR amplifying the pGAPZ-*Ce*DPY19 plasmid^26^ with mutagenic primers in two reactions to give two double-stranded DNA fragments, which were reassembled to give the mutant vector using Gibson Assembly (NEB, E2611L). The mutagenic primer pairs used to make each mutant are provided in Supplementary Tables 2 and 3. These Gibson Assembly reactions were transformed into chemically competent *E. coli* DH5α and transformants selected on low-salt Luria-Bertani media agar plates using zeocin (50 µg ml^−1^) with the exclusion of light. Plasmids from single colonies were prepared for each mutant and verified by Sanger sequencing.

### Integrating *Ce*DPY19 mutants into *Pichia pastoris*

For each mutant, 15 µg of plasmid DNA was linearized overnight using *AvrII* (NEB, R0174S) then purified by ethanol precipitation. This DNA was transformed into electrocompetent *GS115 P. pastoris* cells that had been complemented with a pPIC9K-RNase2 vector to enable methanol-induced expression of human RNAse2 (ref. 26). Transformants were selected for on YPDS agar plates with 100 µg of zeocin at 30 °C for 96 h with the exclusion of light. Colonies for each mutant were restreaked onto fresh yeast extract-peptone-dextrose-sorbitol (YPDS) agar plates with 100 µg of zeocin and grown at 30 °C for 96 h with the exclusion of light. Clones for each mutant were subjected to colony PCR using the primer pairs DPY075/DPY076, DPY077/DPY078 and DPY079/DPY080 (Supplementary Table 2) to confirm integration of the linearized vector into the GAP promoter^26^. Positive clones were used to inoculate YPD medium (5 ml) to produce material to verify mutant *Ce*DPY19 expression by western blot (Supplementary Fig. 2). These cultures were grown to an optical density (OD_600_) of 1.0 and the cells collected by centrifugation (1,500*g*, 30 min, 4 °C). The pellet was resuspended in buffer (10 mM NaPi pH 7.5, 1 M sorbitol, 10 mM EDTA, 100 mM dithiothreitol (DTT)) with lyticase (1 U, SigmaAldrich) and the mixture gently nutated at 37 °C for 1 h. SDS was added to a final concentration of 10% w/v and the mixture gently nutated at 22 °C for 10 min. SDS–PAGE loading buffer was added and the sample held at 37 °C for 10 min (further heating resulted in aggregation of the hydrophobic *Ce*DPY19 protein). Western blot analyses of these samples were performed with M2 anti-FLAG mouse IgG1 (1:5,000, SigmaAldrich, F3165) as the primary antibody and goat antimouse horseradish peroxidase conjugate (1:10,000, ThermoFisher, 62–6520) as the secondary antibody. Membranes were imaged using a ChemiDoc System (Bio-Rad) and processed using Image Lab v.6.1 (Bio-Rad).

### Isolation and digestion of RNase2 coexpressed with *Ce*DPY19 mutants

Each yeast strain harboring a *Ce*DPY19 mutant was used to inoculate BMGY medium (10 ml) and the cultures grown at 30 °C and 225 rpm for 20 h. These were centrifuged (1,500*g*, 10 min, 4 °C), the supernatant discarded and the cell pellet resuspended in BMMY medium to induce expression of RNase2. Cultures were grown at 30 °C and 225 rpm for 20 h, centrifuged (4,000*g*, 30 min, 4 °C) and the supernatant collected. The supernatant was adjusted to be buffered with 50 mM Tris-HCl pH 7.5, 200 mM NaCl and 1 mM EDTA. Protease inhibitor cocktail (Roche, cOmplete EDTA-free) and 0.02% sodium azide were added to the buffered supernatant and the sample filtered (0.22 µm). A 50% slurry (75 µl) of anti-FLAG M2-affinity gel (SigmaAldrich, A2220) was added to capture the secreted RNase2 and the samples nutated overnight at 4 °C. The affinity gel was pelleted by centrifugation (500*g*, 15 min, 4 °C) and the supernatant decanted. The gel was transferred to a spin-cup and washed three times with 500 µl of 50 mM Tris-HCl pH 7.5 and 200 mM NaCl. The spin-cup containing the gel was transferred to a fresh 1.5-ml microcentrifuge tube and 500 µl of elution buffer (50 mM Tris-HCl pH 7.5, 100 mM NaCl, 5% SDS) added. These samples were incubated at 22 °C for 10 min then 85 °C for 5 min before elution of the purified RNase2 in SDS solution by centrifugation (3,000*g*, 3 min). These protein samples were reduced by the addition of DTT to a final concentration of 20 mM with incubation in a ThermoMixer (Eppendorf) at 95 °C and 750 rpm for 15 min. After cooling, iodoacetamide was added to a final concentration of 100 mM and the sample incubated for 30 min at 22 °C with the exclusion of light. These alkylation reactions were quenched by the addition of DTT to a final concentration of 200 mM and incubation at 22 °C for 10 min. These reduced and alkylated samples were acidified with phosphoric acid to a final concentration of 1.2% and then diluted with 6 volumes of S-TRAP binding buffer (100 mM TEAB pH 7.55, 90% methanol). These samples were applied to S-TRAP mini columns (ProtiFi) and washed with S-TRAP binding buffer (4 × 400 µl). The captured protein was digested on-column using sequencing-grade Glu-C (Promega, V1651) in 125 µl 100 mM NH_4_HCO_3_ buffer pH 7.8 at 37 °C for 16 h. Digested peptides were eluted from the column by centrifugation (4,000*g*, 1 min) after each addition of: 80 µl 100 mM NH_4_HCO_3_ pH 7.8; 80 µl 0.2% HCO_2_H in H_2_O, then 0.2% HCO_2_H in 50% MeCN. The combined eluates were lyophilized. To clean up the samples for LC–MS analysis, each sample was resuspended in H_2_O with 0.1% HCO_2_H and 2% MeCN, captured on C18 stage tips, eluted using 0.1% HCO_2_H in H_2_O:MeCN (1:4), then dried and stored at −20 °C.

### LC–MS analysis of digested RNase2

Samples were resuspended in buffer A* (0.1% TFA, 2% MeCN) and separated using a two-column chromatography set-up composed of a PepMap100 C18 20 mm × 75 μm trap and a PepMap C18 500 mm × 75 μm analytical column (ThermoFisher) coupled to an Orbitrap Exploris 480 Mass Spectrometer (ThermoFisher). A 65 min gradient was run for each sample with loading onto the trap column at 6 μl min^−1^ for 6 min using buffer A (2% DMSO, 0.1% HCO_2_H), followed by separation on the analytical column by altering the buffer composition from 3% buffer B (2% DMSO, 78% MeCN, 0.1% HCO_2_H) to 23% buffer B over 29 min; then from 23% buffer B to 40% buffer B over 10 min; then from 40% buffer B to 80% buffer B over 5 min; then holding at 80% buffer B for 5 min; then dropping to 3% buffer B over 1 min and holding at this value for another 9 min. The Orbitrap Exploris 480 Mass Spectrometer was operated in a hybrid data-dependent and independent manner switching between 2.5 s of data-dependent acquisition and roughly 0.5 s of parallel reaction scans for specific peptides of interest. For the data-dependent acquisition, a single Orbitrap MS scan (300–1,800 *m/z*; maximum injection time 25 ms; AGC 300%; resolution 120,000) was undertaken followed by MS2 HCD scans of precursors (maximum injection time 80 ms; AGC 400%; resolution 30,000 and stepped normalized collisional energy of 20, 30 and 40%) for up to 2 s. For parallel reaction monitoring, the ions 1,144.0522, 763.0372, 1,225.0072, 817.0539, 1,306.1022 and 871.0706 corresponding to the +2/+3 charge states of the (glyco)peptide NLYFQGKPPQFTWAQWFE in the nonglycosylated, singly glycosylated and doubly glycosylated states were monitored. Parallel reaction scans were undertaken using a maximum injection time of 80 ms, AGC 500%, resolution 45,000 and stepped normalized collisional energy of 25, 30 and 40%.

### Occupancy analysis of RNAse glycopeptides

To quantify the relative level of *C*-glycosylation within samples, extracted ion chromatograms of the monoisotopic peaks of the +2 charge states were extracted (±10 ppm) using Freestyle Viewer v.1.7 SP1 (Thermo Fisher Scientific). Peaks were processed with a 15-point Gaussian smooth and the area under the curve calculated. The resulting areas were used to calculate the relative abundance of peptide species, with occupation rates determined as a percentage of the total ion current of the compared peptide species. The resulting MS data and search results have been deposited into the PRIDE ProteomeXchange Consortium repository (http://www.proteomexchange.org/). These can be accessed with the identifier PXD032391 using the username reviewer_pxd032391@ebi.ac.uk and password sItWEoNT.

### Data presentation

Figures of structural representations were prepared in PyMol (Schroedinger Inc.), UCSF Chimera (https://www.cgl.ucsf.edu/chimera/) and UCSF ChimeraX (https://www.cgl.ucsf.edu/chimerax/). Graphs were prepared in Prism v.9 (GraphPad Software Inc.). Protein sequence alignments were generated by Clustal Omega^58^ and were depicted using Jalview (https://www.jalview.org/).

### Statistics and reproducibility

Unless otherwise stated, all described in vitro *Ce*DPY19 assays and Tricine gel-based analyses were conducted once as shown in the figures. All in vivo assays were performed as independent triplicates. Depicted ‘representative micrographs’ were chosen randomly but are representative of all micrographs that were visually observed during the respective cryo-EM data collections.

### Reporting summary

Further information on research design is available in the Nature Portfolio Reporting Summary linked to this article.

## Online content

Any methods, additional references, Nature Portfolio reporting summaries, source data, extended data, supplementary information, acknowledgements, peer review information; details of author contributions and competing interests; and statements of data and code availability are available at 10.1038/s41589-022-01219-9.

## Supplementary information

Supplementary Information Supplementary Figs. 1 and 2, Tables 1–3, Methods and Data.

Reporting Summary

## Data Availability

Atomic coordinates of the CeDPY19 models have been deposited in RCSB Protein Data Bank under accession numbers 7ZLH (apo), 7ZLH (peptide-bound), 7ZLI (Dol25-P-Man-bound) and 7ZLJ (Dol25-P-*C*-Man- and peptide-bound). The three-dimensional cryo-EM maps were deposited in the Electron Microscopy Data Bank under accession numbers EMD-14780 (apo), EMD-14779 (peptide-bound), EMD-14781 (Dol25-P-Man-bound) and EMD-14782 (Dol25-P-*C*-Man- and peptide-bound). MS data to quantitate tryptophan *C*-mannosyaltion on RNAse2 has been deposited to the PRIDE proteomics repository under the accession number: PXD032391 using the username reviewer_pxd032391@ebi.ac.uk and password sItWEoNT. Source data are provided with this paper.

## Source data

Source Data Fig. 1 Unprocessed Tricine–SDS–PAGE, raw data of chromatogram and unprocessed Tricine–SDS–PAGE.
Source Data Fig. 4 Raw data used to generate the graph.
Source Data Extended Data Fig. 1 Unprocessed Tricine–SDS–PAGE. The same gel was used to generate Fig. 1b. Raw data of the chromatogram.
Source Data Extended Data Fig. 2 Raw data of chromatogram, unprocessed SDS–PAGE and input data of graph.
Source Data Extended Data Fig. 5 Input data of graph.
