# Supplementary material for: Structure, sequon recognition and mechanism of tryptophan C-mannosyltransferase
Source: Nat Chem Biol. 2023 Jan 5;19(5):575–84. doi: 10.1038/s41589-022-01219-9 (PMC10154233; doi:10.1038/s41589-022-01219-9)

nature chemical biology

Article

https://doi.org/10.1038/s41589-022-01219-9

Structure, sequon recognition and mechanism of tryptophan *C*-mannosyltransferase

In the format provided by the authors and unedited

Supplementary Information

Structure, sequon recognition, and mechanism of tryptophan C-mannosyltransferase

Joël S. Bloch[1,7], Alan John[2,3], Runyu Mao[2,3], Somnath Mukherjee[4], Jérémy Boilevin[5], Rossitza N. Irobalieva[1], Tamis Darbre[5], Nichollas E. Scott[6], Jean-Louis Reymond[5], Anthony A. Kossiakoff[4], Ethan D. Goddard-Borger[2,3]*, Kaspar P. Locher[1]*

[1]Institute of Molecular Biology and Biophysics, ETH Zürich, Otto-Stern-Weg 5, 8093 Zürich, Switzerland.
[2]The Walter and Eliza Hall Institute of Medical Research, Parkville, Victoria 3052, Australia.
[3]Department of Medical Biology, University of Melbourne, Parkville, Victoria 3052, Australia.
[4]Department of Biochemistry and Molecular Biology, University of Chicago, 900 East 57th Street, Chicago, IL 60637, USA.
[5]Department of Chemistry, Biochemistry and Pharmaceutical Sciences, University of Bern, Freiestrasse 3, 3012 Bern, Switzerland.
[6]Department of Microbiology and Immunology, University of Melbourne at the Peter Doherty Institute for Infection and Immunity, Parkville, Victoria 3010, Australia.
[7]Present address: Laboratory of Molecular Neurobiology and Biophysics and Howard Hughes Medical Institute, The Rockefeller University, 1230 York Avenue, New York, NY 10065, USA.

*Correspondence: goddard-borger.e@wehi.edu.au (E.D.G.-B.), locher@mol.biol.ethz.ch (K.P.L.)

Supplementary Figures:

Supplementary Figure 1: Map quality of *Ce*DPY19 apo structure.

Supplementary Figure 2: Western blot analysis of CeDPY19 mutant expression for in vivo glycosylation assays.

Supplementary Tables:

Supplementary Table 1: Cryo-EM data collection, refinement, and validation statistics.

Supplementary Table 2: Oligonucleotide primer sequences used in this study. (Also provided as separate Spreadsheet)

Supplementary Table 3: Oligonucleotide pairs used to mutagenize pGAPZ-CeDPY19.

Supplementary Note:

Synthesis of Dol-C-P-Man.

LC-MS analysis of glycopeptides.

Analytical chemistry and chemical compounds.

Source Data for Supplementary Figure 2.

Supplementary Figures

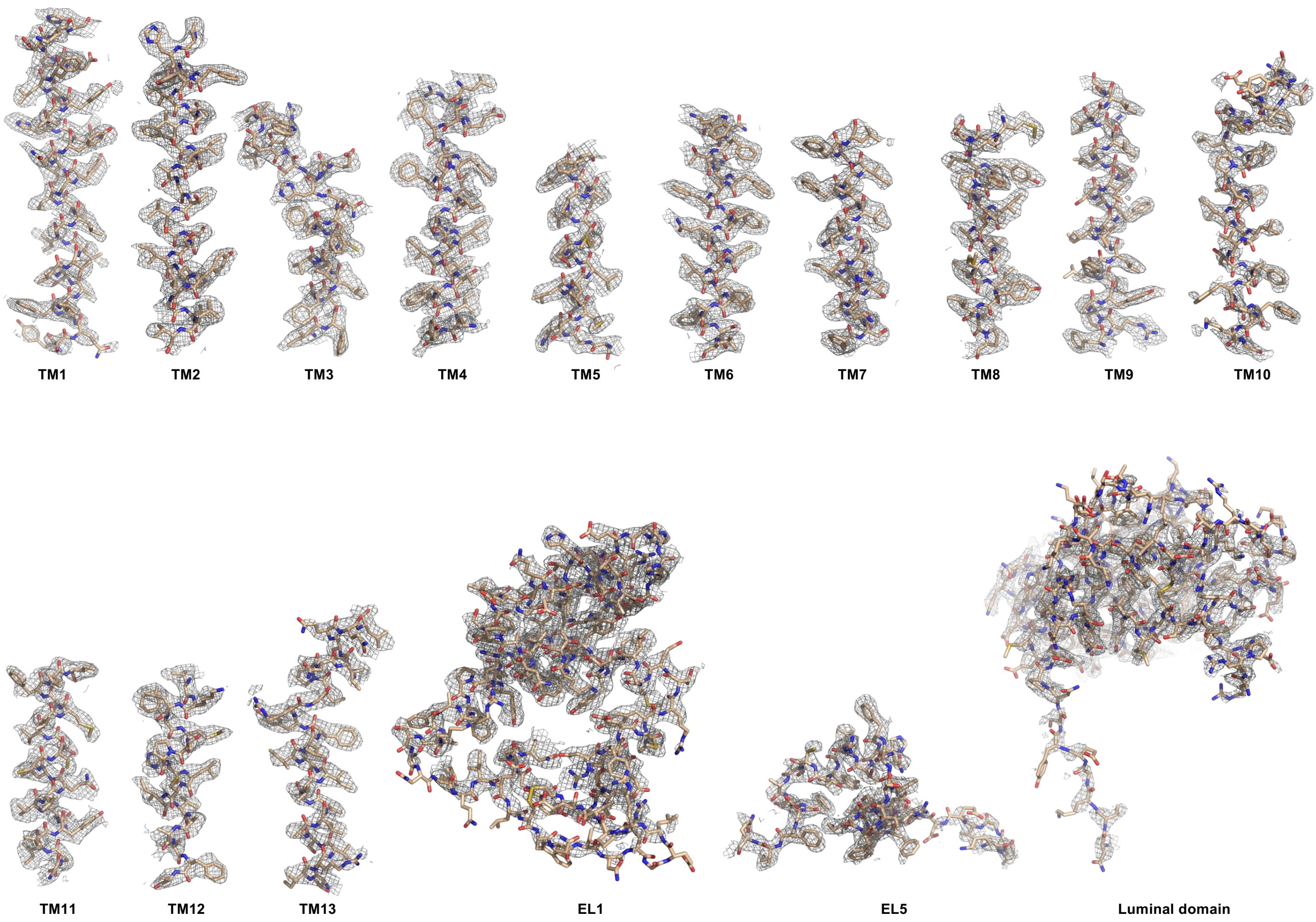

Supplementary Figure 1 | Map quality of *Ce*DPY19 apo structure. *Ce*DPY19 is depicted in stick representation. Maps are displayed at a contour level of 8.0 r.m.s.d. and were carved at 2.2 Å.

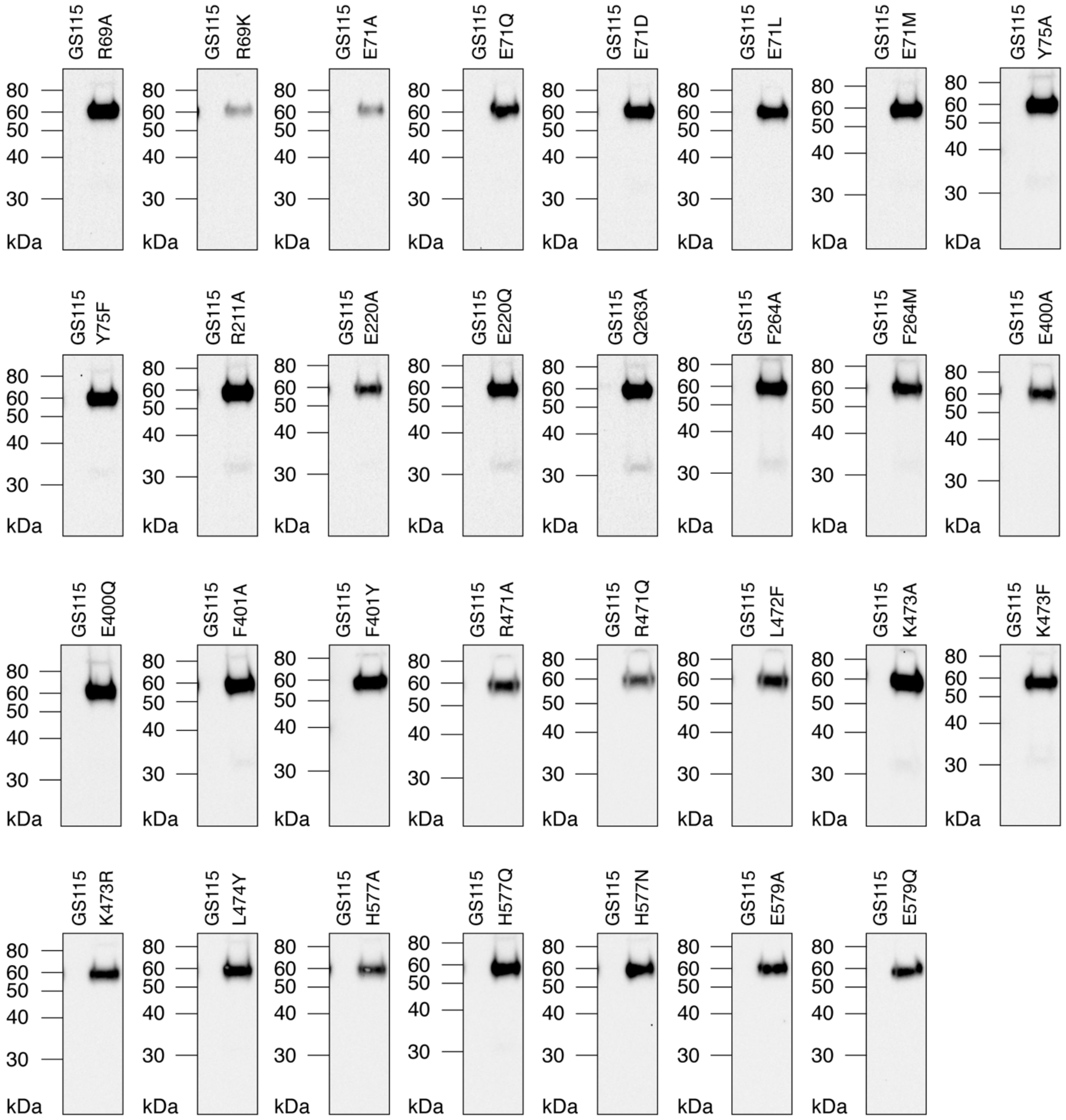

Supplementary Figure 2 | Western blot analysis of *Ce*DPY19 mutant expression for in vivo glycosylation assays. C-terminally FLAG3-tagged *Ce*DPY19 mutants were expressed in *P. pastoris* and the corresponding expression levels were assessed by Western-Blot analysis of the lysates using an M2 anti-FLAG mouse IgG1 (1:5000, SigmaAldrich, F3165) as the primary antibody and goat anti-mouse horseradish peroxidase conjugate (1:10000, ThermoFisher, 62-6520) as the secondary antibody.

Supplementary Tables:

Supplementary Table 1 | Cryo-EM data collection, refinement, and validation statistics.

	#1 CeDPY19–CMT2-Fab–anti-Fab Nb apo structure (EMDB-14780) (PDB ID 7ZLH)	#2 CeDPY19–CMT2-Fab–anti-Fab Nb + acceptor peptide (EMDB-14779) (PDB ID 7ZLG)	#3 CeDPY19–CMT2-Fab–anti-Fab Nb + Dol25-P-Man (EMDB-14781) (PDB ID 7ZLI)	#4 CeDPY19–CMT2-Fab–anti-Fab Nb + acceptor peptide + Dol25-P-C-Man (EMDB-14782) (PDB ID 7ZLJ)
**Data collection and processing**				
Magnification	130,000	130,000	130,000	130,000
Voltage (kV)	300	300	300	300
Electron exposure per frame (e–/Å^2)	1.21	1.21	1.21	1.21
Defocus range (μm)	-0.6 to -0.8	-0.6 to -0.8	-0.6 to -0.8	-0.6 to -0.8
Pixel size (Å)	0.66	0.66	0.66	0.66
Symmetry imposed	C1	C1	C1	C1
Initial particle images (no.)	8,937,261	3,876,382	2,543,900	4,309,649
Final particle images (no.)	384,830	324,852	301,020	57,289
Map resolution (Å)	2.75	2.72	2.99	3.63
FSC threshold	0.143	0.143	0.143	0.143
Map resolution range (Å)	2.70-3.20	2.65-3.15	2.95-3.45	3.50-4.10
**Refinement**				
Initial model used (PDB code)	6ANI (Fab+Nb)	6ANI (Fab+Nb)	6ANI (Fab+Nb)	6ANI (Fab+Nb)
Model resolution (Å)	2.44	2.30	2.94	3.58
FSC threshold	0.143	0.143	0.143	0.143
Model resolution range (Å)	2.44-2.59	2.30-2.30	2.94-2.99	3.65-3.86
Map sharpening *B* factor (Å^2)	-89.7	-82.7	-120.7	-107.1
Model composition				
Non-hydrogen atoms	9480	9618	9636	9715
Protein residues	1211	1226	1218	1226
Ligands	0	0 (ligand = peptide)	1	1 (1 ligand = peptide)
B factors (Å^2)				
Protein	89.93	109.82	63.87	51.52
Ligand	n/a	n/a	42.22	19.95
R.m.s. deviations				
Bond lengths (Å)	0.004	0.004	0.007	0.005
Bond angles (°)	0.807	0.716	0.970	0.787
Validation				
MolProbity score	1.90	1.72	2.37	2.32
Clashscore	9.46	7.08	27.21	31.81
Poor rotamers (%)	0.38	0.66	0.96	0.00
Ramachandran plot				
Favored (%)	93.99	95.23	93.05	95.15
Allowed (%)	5.67	4.36	6.54	4.77
Disallowed (%)	0.33	0.41	0.41	0.08

Supplementary Table 2 | Oligonucleotide primer sequences used in this study.

primer	sequence
DPY001	GTCCTGGACCGCGCTGATGAACAGGGTCACGTC
DPY002	GACGTGACCCTGTTCATCAGCGCGGTCCAGGAC
DPY003	CTTCGAAAGAGAGATGGCTTACGCAACTGAGATGGGTTTGTACTACTC
DPY004	CTTCGAAAGAGAGATGGCTTACAAAACTGAGATGGGTTTGTACTACTC
DPY005	CGAAAGAGAGATGGCTTACAGAGCTGAGATGGGTTTGTACTACTCCTACTAC
DPY006	CGAAAGAGAGATGGCTTACAGAGGTGAGATGGGTTTGTACTACTCCTACTAC
DPY007	GAAAGAGAGATGGCTTACAGAACTGCGATGGGTTTGTACTACTCCTACTAC
DPY008	GAAAGAGAGATGGCTTACAGAACTCAGATGGGTTTGTACTACTCCTACTAC
DPY009	GAAAGAGAGATGGCTTACAGAACTGACATGGGTTTGTACTACTCCTACTAC
DPY010	GAAAGAGAGATGGCTTACAGAACTCTGATGGGTTTGTACTACTCCTACTAC
DPY011	GAAAGAGAGATGGCTTACAGAACTATGATGGGTTTGTACTACTCCTACTAC
DPY012	GCTTACAGAACTGAGATGGGTTTGTACTACTCCTACTACAAGACTATCATCAACG
DPY013	GCTTACAGAACTGAGATGGGTTTGTTCTACTCCTACTACAAGACTATCATCAACG
DPY014	CAACCACGGTGAGGCTACTGCGGTTCAATGGACTCCACCATTG
DPY015	CAATGGACTCCACCATTGAGAGCGTCCTTCGCTTTCCCATTCATC
DPY016	CAATGGACTCCACCATTGAGACAGTCCTTCGCTTTCCCATTCATC
DPY017	GTTCCAGCTTTGTTGTTCTGGGCGTTCACTCAGTTCGCTTTCTTC
DPY018	CAGCTTTGTTGTTCTGGCAGGCCACTCAGTTCGCTTTCTTCAC
DPY019	CAGCTTTGTTGTTCTGGCAGATGACTCAGTTCGCTTTCTTCAC
DPY020	CCTTCGCTAACTTCCACACTAGATTGGCCACTTGTTCCGCTGAGTTCGAC

DPY021	CCTTCGCTAACTTCCACACTAGATTGTTCACTTGTTCCGCTGAGTTCGAC
DPY022	CTAGATTGTACACTTGTTCCGCTGCGTTCGACTTCATCCAGTACTCC
DPY023	CTAGATTGTACACTTGTTCCGCTCAGTTCGACTTCATCCAGTACTCC
DPY024	GTACACTTGTTCCGCTGAGGCCGACTTCATCCAGTACTCCACTATCG
DPY025	GTACACTTGTTCCGCTGAGTACGACTTCATCCAGTACTCCACTATCG
DPY026	CCACTGTTATGGCTTTTTTGATCATGGCGTTGAAGTTGTTCATGACTCC
DPY027	CCACTGTTATGGCTTTTTTGATCATGCAATTGAAGTTGTTCATGACTCCACACTTG
DPY028	CCACTGTTATGGCTTTTTTGATCATGAAATTGAAGTTGTTCATGACTCCACACTTG
DPY029	GTTATGGCTTTTTTGATCATGAGATTCAAGTTGTTCATGACTCCACAC
DPY030	GTTATGGCTTTTTTGATCATGAGATTGGCGTTGTTCATGACTCCACACTTG
DPY031	GTTATGGCTTTTTTGATCATGAGATTGACGTTGTTCATGACTCCACACTTG
DPY032	GTTATGGCTTTTTTGATCATGAGATTGAGGTTGTTCATGACTCCACACTTG
DPY033	GCTTTTTTGATCATGAGATTGAAGTACTTCATGACTCCACACTTGTG
DPY034	GACCAATCGTTAACCACCCTGCCTACGAACACGTTGGTATCAGAG
DPY035	GACCAATCGTTAACCACCCTCAGTACGAACACGTTGGTATCAGAG
DPY036	GACCAATCGTTAACCACCCTAACTACGAACACGTTGGTATCAGAG
DPY037	GACCAATCGTTAACCACCCTCACTACGCACACGTTGGTATCAGAGAGAGAACTTTG
DPY038	GACCAATCGTTAACCACCCTCACTACCAACACGTTGGTATCAGAGAGAGAACTTTG
DPY039	GAGTAGTACAAACCCATCTCAGTTGCGTAAGCCATCTCTCTTTCGAAG

DPY040	GAGTAGTACAAACCCATCTCAGTTTTGTAAGCCATCTCTCTTTCGAAG
DPY041	GTAGTAGGAGTAGTACAAACCCATCTCAGCTCTGTAAGCCATCTCTCTTTCG
DPY042	GTAGTAGGAGTAGTACAAACCCATCTCACCTCTGTAAGCCATCTCTCTTTCG
DPY043	GTAGTAGGAGTAGTACAAACCCATCGCAGTTCTGTAAGCCATCTCTCTTTC
DPY044	GTAGTAGGAGTAGTACAAACCCATCTGAGTTCTGTAAGCCATCTCTCTTTC
DPY045	GTAGTAGGAGTAGTACAAACCCATGTCAGTTCTGTAAGCCATCTCTCTTTC
DPY046	GTAGTAGGAGTAGTACAAACCCATCAGAGTTCTGTAAGCCATCTCTCTTTC
DPY047	GTAGTAGGAGTAGTACAAACCCATCATAGTTCTGTAAGCCATCTCTCTTTC
DPY048	CGTTGATGATAGTCTTGTAGTAGGAGTAGTACAAACCCATCTCAGTTCTGTAAGC
DPY049	CGTTGATGATAGTCTTGTAGTAGGAGTAGAACAAACCCATCTCAGTTCTGTAAGC
DPY050	CAATGGTGGAGTCCATTGAACCGCAGTAGCCTCACCGTGGTTG
DPY051	GATGAATGGGAAAGCGAAGGACGCTCTCAATGGTGGAGTCCATTG
DPY052	GATGAATGGGAAAGCGAAGGACTGTCTCAATGGTGGAGTCCATTG
DPY053	GAAGAAAGCGAACTGAGTGAACGCCCAGAACAACAAAGCTGGAAC
DPY054	GTGAAGAAAGCGAACTGAGTGGCCTGCCAGAACAACAAAGCTG
DPY055	GTGAAGAAAGCGAACTGAGTCATCTGCCAGAACAACAAAGCTG
DPY056	GTCGAACTCAGCGGAACAAGTGGCCAATCTAGTGTGGAAGTTAGCGAAGG
DPY057	GTCGAACTCAGCGGAACAAGTGAACAATCTAGTGTGGAAGTTAGCGAAGG
DPY058	GGAGTACTGGATGAAGTCGAACGCAGCGGAACAAGTGTACAATCTAG

DPY059	GGAGTACTGGATGAAGTCGAACTGAGCGGAACAAGTGTACAATCTAG
DPY060	CGATAGTGGAGTACTGGATGAAGTCGGCCTCAGCGGAACAAGTGTAC
DPY061	CGATAGTGGAGTACTGGATGAAGTCGTACTCAGCGGAACAAGTGTAC
DPY062	GGAGTCATGAACAACTTCAACGCCATGATCAAAAAAGCCATAACAGTGG
DPY063	CAAGTGTGGAGTCATGAACAACTTCAATTGCATGATCAAAAAAGCCATAACAGTGG
DPY064	CAAGTGTGGAGTCATGAACAACTTCAATTTCATGATCAAAAAAGCCATAACAGTGG
DPY065	GTGTGGAGTCATGAACAACTTGAATCTCATGATCAAAAAAGCCATAAC
DPY066	CAAGTGTGGAGTCATGAACAACGCCAATCTCATGATCAAAAAAGCCATAAC
DPY067	CAAGTGTGGAGTCATGAACAACGTCAATCTCATGATCAAAAAAGCCATAAC
DPY068	CAAGTGTGGAGTCATGAACAACCTCAATCTCATGATCAAAAAAGCCATAAC
DPY069	CACAAGTGTGGAGTCATGAAGTACTTCAATCTCATGATCAAAAAAGC
DPY070	CTCTGATACCAACGTGTTCGTAGGCAGGGTGGTTAACGATTGGTC
DPY071	CTCTGATACCAACGTGTTCGTACTGAGGGTGGTTAACGATTGGTC
DPY072	CTCTGATACCAACGTGTTCGTAGTTAGGGTGGTTAACGATTGGTC
DPY073	CAAAGTTCTCTCTCTGATACCAACGTGTGCGTAGTGAGGGTGGTTAACGATTGGTC
DPY074	CAAAGTTCTCTCTCTGATACCAACGTGTTGGTAGTGAGGGTGGTTAACGATTGGTC
DPY075	GATTTTGGTCATGAGATCAG
DPY076	TCTGGAATAACCTTACCG
DPY077	GATCTCAAGAAGATCCTTTGATC
DPY078	CCATTCATCATCGGTCACATTG
DPY079	CTTAAAATTGCCCCTTTCAC

DPY080	CAATACCCTCACAGGATTC

Supplementary Table 3 Oligonucleotide pairs used to mutagenize pGAPZ-CeDPY19.

*Ce*DPY19 mutant	primer pair 1	primer pair 2
R69A	DPY001 + DPY003	DPY002 + DPY039
R69K	DPY001 + DPY004	DPY002 + DPY040
T70A	DPY001 + DPY005	DPY002 + DPY041
T70V	DPY001 + DPY006	DPY002 + DPY042
E71A	DPY001 + DPY007	DPY002 + DPY043
E71Q	DPY001 + DPY008	DPY002 + DPY044
E71D	DPY001 + DPY009	DPY002 + DPY045
E71L	DPY001 + DPY010	DPY002 + DPY046
E71M	DPY001 + DPY011	DPY002 + DPY047
Y75A	DPY001 + DPY012	DPY002 + DPY048
Y75F	DPY001 + DPY013	DPY002 + DPY049
R211A	DPY001 + DPY014	DPY002 + DPY050
E220A	DPY001 + DPY015	DPY002 + DPY051
E220Q	DPY001 + DPY016	DPY002 + DPY052
Q263A	DPY001 + DPY017	DPY002 + DPY053
F264A	DPY001 + DPY018	DPY002 + DPY054
F264M	DPY001 + DPY019	DPY002 + DPY055
Y395A	DPY001 + DPY020	DPY002 + DPY056
Y395F	DPY001 + DPY021	DPY002 + DPY057
E400A	DPY001 + DPY022	DPY002 + DPY058
E400Q	DPY001 + DPY023	DPY002 + DPY059
F401A	DPY001 + DPY024	DPY002 + DPY060
F401Y	DPY001 + DPY025	DPY002 + DPY061
R471A	DPY001 + DPY026	DPY002 + DPY062
R471Q	DPY001 + DPY027	DPY002 + DPY063
R471K	DPY001 + DPY028	DPY002 + DPY064
L472F	DPY001 + DPY029	DPY002 + DPY065
K473A	DPY001 + DPY030	DPY002 + DPY066
K473R	DPY001 + DPY032	DPY002 + DPY068
L474Y	DPY001 + DPY033	DPY002 + DPY069

H577A	DPY001 + DPY034	DPY002 + DPY070
H577Q	DPY001 + DPY035	DPY002 + DPY071
H577N	DPY001 + DPY036	DPY002 + DPY072

Supplementary Note

Synthesis of Dol25-P-C-Man

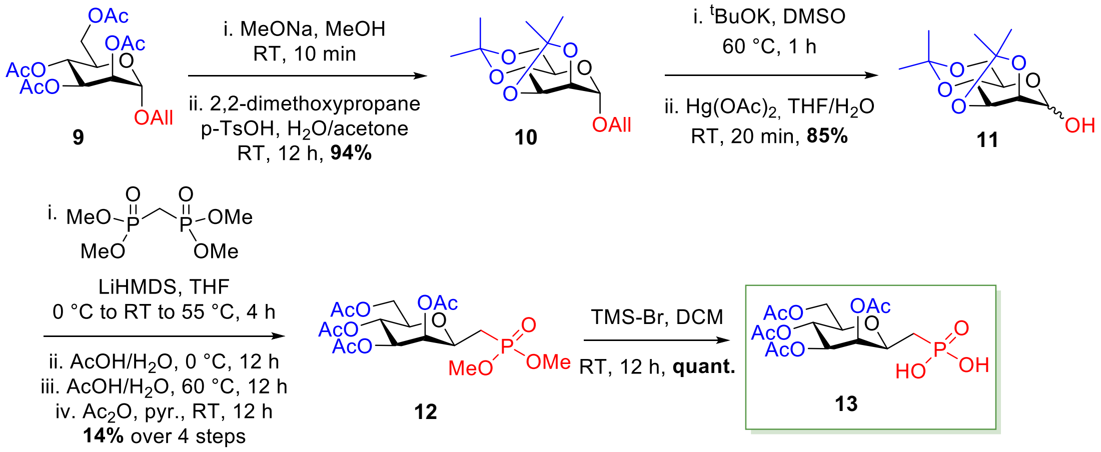

Scheme 1: Nine-step synthesis of mannose β-phosphonate **13**

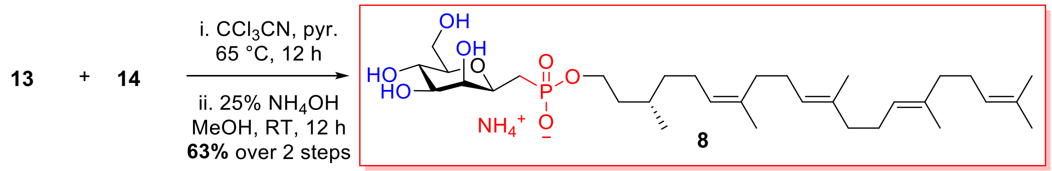

Scheme 2: Man-CP-C25-Farnesylcitronellyl **8** phosphonate by trichloroacetonitrile activation of Man β-phosphonate **13** and S_N2 reaction with lipid alcohol **14**.

β-D-Mannosyl phosphonate **13** was synthesized from allyl 2,3,4,6-tetra-O-acetyl-α-D-mannopyranoside **9** in a nine-step sequence based on a previously described method[1]. Notably, installing isopropylidene protecting groups was essential to achieve a high degree of anomeric control during the key Horner-Wadsworth-Emmons (HWE) phosphonate insertion reaction. The α-anomer was readily separated after cleavage of the less stable 4,6-*O*-isopropylidene protecting group (**Scheme 1**).

Finally, trichloroacetonitrile activation of glycosyl donor **13**, coupling with (*S*)-farnesylcitronellol[2] **14** and global deprotection as previously reported[3] allowed the isolation of Man-CP-C25-Farnesylcitronellyl **8** (**Scheme 2**).

1. Borodkin, V. S.; Ferguson, M. A. J.; Nikolaev, A. V. *Tetrahedron Lett.* **2001**, *42* (31), 5305.
2. Ramírez, A. S.; Boilevin, J.; Biswas, R.; Gan, B. H.; Janser, D.; Aebi, M.; Darbre, T.; Reymond, J. L.; Locher, K. P. *Glycobiology* **2017**, *27* (6), 525.
3. Bloch, J. S.; Pescuillesi, G.; Boilevin, J.; Nosol, K.; Irobalieva, R. N.; Darbre, T.; Aebi, M.; Kossiakoff, A. A.; Reymond, J. L.; Locher, K. P. *Nature* **2020**, *579*, 443.

(3aS,4S,5aR,9aR,9bS)-2,2,8,8-tetramethyl-4-(((E)-prop-1-en-1-yl)oxy)hexahydro-[1,3]dioxolo[4',5':4,5]pyrano[3,2-d][1,3]dioxine 10

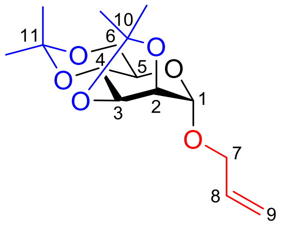

9 (4.50 g, 11.59 mmol, 1.00 eq) was dissolved in 25 mL MeOH and sodium methoxide (2.65 mL, 11.59 mmol, 1.00 eq, 25 wt% in MeOH) was added. The reaction mixture was sonicated at RT for 10 min, neutralized with Amberlyst IR-15 ion exchange resin and concentrated to dryness. The residue was dissolved in 40 mL dry acetone followed by the addition of 2,2-dimethoxypropane (11.40 mL, 92.70 mmol, 10.00 eq) and *p*-toluenesulfonic acid monohydrate (441 mg, 2.32 mmol, 0.20 eq) under an Ar atmosphere at RT. The reaction mixture was stirred overnight, diluted in EtOAc, washed with saturated $NaHCO_3$, brine, dried over Na_2SO_4, filtered and concentrated *in vacuo*. The residue was purified by flash column chromatography on silica gel (DCM to DCM/MeOH 99:1, Rf = 0.11 in DCM) to yield **2** (3.26 g, 10.85 mmol, 94%) as a colorless oil. ^1H NMR (400 MHz, $CDCl_3$) δ = 5.84-5.94 (m, 1H, H-8), 5.29 (dd, *J* = 17.2 Hz, 1.6 Hz, 1H, H-9a), 5.22 (dd, *J* = 10.4 Hz, 1.2 Hz, 1H, H-9b), 5.06 (s, 1H, H-1), 4.14-4.20 (m, 3H, H-7a, H-2, H-3), 3.96-4.01 (m, 1H, H-7b), 3.85-3.89 (m, 1H, H-6a), 3.72-3.78 (m, 2H, H-6b, H-4), 3.57-3.63 (m, 1H, H-5), 1.54, 1.51, 1.42, 1.35 (4xs, 4x3H, 4xMe). ^{13}C NMR (101 MHz, $CDCl_3$) δ = 133.6 (s, C-8), 118.1 (s, C-9), 109.6 (s, C-10), 99.8 (s, C-11), 97.1 (s, C-1), 76.3 (s, C-2), 75.0 (s, C-3), 72.9 (s, C-4), 68.3 (s, C-7), 62.2 (s, C-6), 61.5 (s, C-5), 29.2, 28.3, 26.3, 18.9 (4xs, 4xMe). ESI-MS (+) *m/z* calculated 301.16 (M+[H^+]), found 301.17 for $C_{15}H_{25}O_6^+$.

(3aS,5aR,9aR,9bS)-2,2,8,8-tetramethylhexahydro-[1,3]dioxolo[4',5':4,5]pyrano[3,2-d][1,3]dioxin-4-ol 11

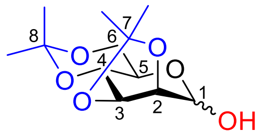

To a solution of **10** (610 mg, 2.03 mmol, 1.00 eq) in 5.5 mL DMSO was added potassium *tert*-butylate (912 mg, 8.12 mmol, 4.00 eq) at RT under an Ar atmosphere. The reaction mixture was then stirred for 1 h at 60 °C and quenched by addition of water. The aqueous phase was extracted 4 times with EtOAc and the combined organic layers were washed once with water, dried over Na_2SO_4, filtered and concentrated to dryness. The residue was dissolved in 11 mL of a mixture of THF/H_2O 9:1 and $Hg(OAc)_2$ (1.29 g, 4.06 mmol, 2.00 eq) was added portionwise. The reaction mixture was stirred for 20 min at RT, filtered on celite and concentrated. The residue was dissolved in EtOAc, washed with water and brine, dried over Na_2SO_4, filtered and concentrated to dryness to yield **11** (450 mg, 1.73 mmol, 85%) as a colorless solid as an alpha/beta mixture which was used in the next step without further purification. ^1H NMR alpha product (400 MHz, $CDCl_3$) δ = 5.44 (d, *J* = 3.3 Hz, 4.17-4.23 (m, 2H, H-2, H-3), 3.86-3.89 (m, 1H, H-6a), 3.74-3.83 (m, 3H, H-4, H-5, H-6b), 2.57 (d, *J* = 3.3 Hz, 1H, OH), 1.55, 1.52, 1.43, 1.36 (4xs, 4x3H, 4xMe). ^{13}C NMR (101 MHz, $CDCl_3$) δ = 109.7 (s, C-7), 99.9 (s, C-8), 93.1 (s, C-1), 76.3 (s, C-2), 74.9 (s, C-3), 72.8 (s, C-4), 62.3 (s, C-6), 61.9 (s, C-5), 29.2, 28.3, 26.3, 19.0 (4xs, 4xMe). ESI-MS (+) *m/z* calculated 261.13 (M+[H^+]), found 261.13 for $C_{12}H_{21}O_6^+$.

(2R,3R,4S,5S,6R)-2-(acetoxymethyl)-6-((dimethoxyphosphoryl)methyl)tetrahydro-2H-pyran-3,4,5-triyl triacetate 12

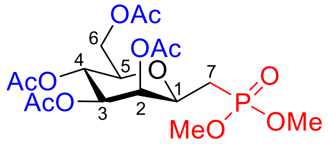

11 (440 mg, 1.69 mmol, 1.00 eq) was added dropwise to a mixture of bis(dimethoxyphosphoryl)methane (432 mg, 1.90 mmol, 1.10 eq) and LiHMDS (1.86 mL, 1.86 mmol, 1.10 eq, 1 M in THF) in 5 mL THF at 0 °C under an Ar atmosphere. The reaction mixture was warmed up to RT over 1 h and heated up to 55 °C for another hour. The reaction mixture was then diluted with EtOAc, washed with saturated $NaHCO_3$ and brine, dried over Na_2SO_4, filtered and concentrated *in vacuo*. The residue was dissolved in 4 mL of a mixture of AcOH/H_2O 4:1 at 0 °C and the reaction mixture was stirred at this temperature overnight, concentrated to dryness and the two anomers were separated by flash column chromatography on silica gel (EtOAc to DCM/MeOH 98:2 to 97:3). The beta anomer was dissolved in 4 mL of a mixture of AcOH/H_2O 4:1 at RT and the reaction mixture was stirred at 65 °C for 2 h and concentrated to dryness. The residue was dissolved in 4 mL of a mixture of Ac_2O/pyridine 1:1, stirred at RT overnight and concentrated to dryness. The residue was purified by flash column chromatography on silica gel (DCM/MeOH 98:2 to 96:4, Rf = 0.20 in DCM/MeOH 98:2) to yield **12** (110 mg, 0.24 mmol, 14%) as a colorless foam. ^{1}H NMR (400 MHz, $CDCl_3$) δ = 5.34 (dd, *J* = 3.2 Hz, 0.8 Hz, 1H, H-2), 5.19 (t, *J* = 10.0 Hz, 1H, H-4), 5.06 (dd, *J* = 10.0 Hz, 3.2 Hz, 1H, H-3), 4.21 (dd, *J* = 12.4 Hz, 2.4 Hz, 1H, H-6a), 4.12 (dd, *J* = 12.4 Hz, 2.4 Hz, 1H, H-6b), 4.04-4.09 (m, 1H, H-1), 3.74 (d, *J* = 8.0 Hz, 3H, OMe), 3.72 (d, *J* = 8.0 Hz, 3H, OMe), 3.68-3.80 (m, 1H, H-5), 2.17, 2.08, 2.04, 1.97 (4xs, 4x3H, 4xOAc), 2.05-2.14 (m, 2H, H-7). ^{13}C NMR (101 MHz, $CDCl_3$) δ = 170.7, 170.4, 170.1, 169.8 (4xs, 4×OC=O), 76.6 (s, C-5), 72.5 (d, *J* = 1.3 Hz, C-1), 72.2 (d, *J* = 1.2 Hz, C-3), 70.1 (d, *J* = 10.4 Hz, C-2), 65.9 (s, C-4), 62.9 (s, C-6), 53.0 (d, *J* = 6.7 Hz, OMe), 52.4 (d, *J* = 6.7 Hz, OMe), 27.5 (d, *J* = 144.8 Hz, C-7), 20.9, 20.8, 20.8, 20.7 (4xs, 4xOC=O$\underline{C}H_3$). ^{31}P NMR (122 MHz, $CDCl_3$) δ = 29.1. ESI-MS (+) *m/z* calculated 455.13 (M+[H^+]), found 455.13 for $C_{17}H_{28}O_{12}P^+$.

(((2R,3S,4S,5R,6R)-3,4,5-triacetoxy-6-(acetoxymethyl)tetrahydro-2H-pyran-2-yl)methyl)phosphonic acid 13

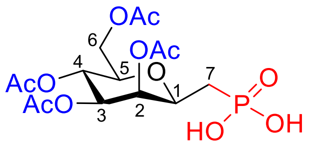

12 (96 mg, 0.21 mmol, 1.00 eq) was dissolved in 2 mL dry DCM and TMSBr (0.56 mL,4.23 mmol, 20.00 eq) was added dropwise at 0 °C under an Ar atmosphere. The reaction mixture was then stirred at RT for 12 h, concentrated *in vacuo*, dissolved in 9 mL acetone and 0.60 mL H_2O was added. The reaction mixture was stirred at RT for 30 min and then concentrated to dryness to yield **13** (90 mg, 0.21 mmol, quant.) as a colorless solid which was used in the next step without further purification.

(2R,3R,4S,5S,6R)-2-(acetoxymethyl)-6-((hydroxy(((S,6Z,10E,14E)-3,7,11,15,19-pentamethylicosa-6,10,14,18-tetraen-1-yl)oxy)phosphoryl)methyl)tetrahydro-2H-pyran-3,4,5-triyl triacetate

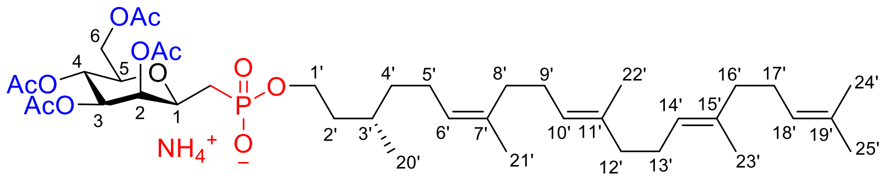

13 (90 mg, 0.21 mmol, 1.00 eq) and **14** (305 mg, 0.84 mmol, 4.00 eq) were dissolved in 4 mL pyridine and trichloroacetonitrile (0.21 mL, 2.11 mmol, 10.00 eq) was added at RT under an Ar atmosphere. The reaction mixture was warmed up to 65 °C, stirred overnight and concentrated to dryness. The residue was purified by flash column chromatography on silica gel basified with ammonium hydroxide 25% solution in water (EtOAc/MeOH 8:2 to 7:3, Rf = 0.28 in EtOAc/MeOH 7:3) to yield **the acetylated Man-CP-*(S)*-Farnesylcitronellyl** (105 mg, 0.13 mmol, 63%) as a colorless sticky solid. ^{1}H NMR (400 MHz, MeOD) δ = 5.44 (d, *J* = 2.4 Hz, 1H, H-2), 5.09-5.20 (m, 6H, H-2, H-3, H-6', H-10', H-14', H-18'), 4.25 (dd, *J* = 12.4 Hz, 1.2 Hz, 1H, H-6a), 4.07-4.11 (m, 2H, H-1, H-6b), 3.92-3.97 (m, 2H, H-1'), 3.76-3.80 (m, 1H, H-5), 2.16 (s, 3H, OAc), 1.95-2.12 (m, 22H, H-7, 2xOAc, H-5', H-8', H-9', H-12', H-13', H-16', H-17'), 1.93 (s, 3H, OAc), 1.67-1.68 (m, 7H, H-2'a, H-21', H-22'), 1.60-1.62 (m, 10H, H-3', H-23', H-24', H-25'), 1.33-1.46 (m, 2H, H-2'b, H-4'a), 1.16-1.19 (m, 1H, H-4'b), 0.92 (d, *J* = 6.4 Hz, 3H, H-20'). ^{13}C NMR (101 MHz, MeOD) δ = 172.4, 172.0, 171.6, 171.4 (4xs, 4xOC=O), 136.0 (s, C-7'), 135.9 (s, C-11'), 135.9 (s, C-15'), 132.0 (s, C-19'), 126.6 (s, C-18'), 125.5 (s, C-14'), 125.5 (s, C-10'), 125.5 (s, C-6'), 77.3 (s, C-5), 74.5 (s, C-1), 73.9 (s, C-3), 71.8 (d, *J* = 7.9 Hz, C-2), 67.2 (s, C-4), 64.1 (d, *J* = 6.3 Hz, C-1'), 63.9 (s, C-6), 40.9 (s, C-8'), 40.8 (s, C-12'), 38.9 (s, C-2'), 38.8 (s, C-9'), 38.6 (s, C-4'), 32.9 (s, C-13'), 30.4 (s, C-3'), 28.3 (d, *J* = 148.9 Hz, C-7), 27.6 (s, C-16'), 27.6 (s, C-5'), 26.4 (s, C-17'), 25.9 (s, C-21'), 23.7 (s, C-22'), 20.7, 20.7, 20.6, 20.6 (4xs, 4xOC=O$\underline{C}H_3$), 19.8 (s, C-20'), 17.8 (s, C-23'), 16.2 (s, C-24'), 16.1 (s, C-25'). ^{31}P NMR (122 MHz, $CDCl_3$) δ = 22.4. ESI-HRMS (-) *m/z* calculated 767.4141 (M-[H^+]), found 767.4166 for $C_{40}H_{64}O_{12}P^-$.

(S,6Z,10E,14E)-3,7,11,15,19-pentamethylicosa-6,10,14,18-tetraen-1-yl (((2R,3S,4S,5S,6R)-3,4,5-trihydroxy-6-(hydroxymethyl)tetrahydro-2H-pyran-2-yl)methyl)phosphonate 8

The acetylated Man-CP-*(S)*-Farnesylcitronellyl (51 mg, 0.08 mmol, 1.00 eq) was dissolved in 5 mL MeOH and an excess of a 25% ammonium hydroxide solution in water was added dropwise (200 eq per function). The reaction mixture was stirred at RT for 12 h. MeOH was removed *in vacuo* and acetamide and ammonium hydroxide were removed at the freeze-dryer to yield **8** (75.50 mg, 0.12 mmol, quant.) as a colorless lyophilisat

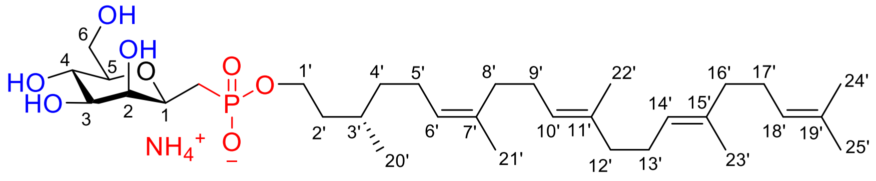

(Rf = 0.50, EtOAc/MeOH 4:6). ^{1}H NMR (400 MHz, MeOD) δ = 5.08-5.14 (m, 4H, H-6', H-10', H-14', H-18'), 3.85-3.94 (m, 3H, H-2, H-1'), 3.78-3.82 (m, 2H, H-1, H-6a), 3.66 (dd, J = 12.0 Hz, 6.0 Hz, 1H, H-6b), 3.45-3.54 (m, 2H, H-3, H-4), 3.20-3.23 (m, 1H, H-5), 1.94-2.12 (m, 16H, H-7, H-5', H-8', H-9', H-12', H-13', H-16', H-17'), 1.67-1.71 (m, 7H, H-2'a, H-21', H-22'), 1.60-1.62 (m, 10H, H-3', H-23', H-24', H-25'), 1.33-1.45 (m, 2H, H-2'b, H-4'a), 1.16-1.20 (m, 1H, H-4'b), 0.92 (d, J = 6.8 Hz, 3H, H-20'). ^{13}C NMR (101 MHz, MeOD) δ = 136.0 (s, C-7'), 135.9 (s, C-11'), 135.9 (s, C-15'), 132.0 (s, C-19'), 126.6 (s, C-18'), 125.5 (s, C-14'), 125.5 (s, C-10'), 125.5 (s, C-6'), 82.1 (s, C-5), 76.5 (s, C-3), 76.3 (s, C-1), 72.9 (d, J = 6.6 Hz, C-2), 68.8 (s, C-4), 63.4 (d, J = 5.9 Hz, C-1'), 63.1 (s, C-6), 40.9 (s, C-8'), 40.8 (s, C-12'), 39.3 (s, C-2'), 39.2 (s, C-9'), 38.8 (s, C-4'), 32.9 (s, C-13'), 30.6 (s, C-3'), 30.4 (d, J = 135.6 Hz, C-7), 27.8 (s, C-16'), 27.6 (s, C-5'), 26.4 (s, C-17'), 25.9 (s, C-21'), 23.7 (s, C-22'), 19.8 (s, C-20'), 17.8 (s, C-23'), 16.1 (s, C-24'), 16.1 (s, C-25'). ^{31}P NMR (122 MHz, $CDCl_3$) δ = 21.4. ESI-HRMS (-) *m/z* calculated 599.3718 (M-[H^+]), found 599.3739 for $C_{32}H_{56}O_8P^-$.

LC-MS analysis of glycopeptides.

2 uL of samples were analyzed on a calibrated Q-Exactive mass spectrometer (Thermo Fischer Scientific) coupled to a nano-Acquity UPLC system (Waters). Peptides were resuspended in 2.5% acetonitrile with 0.1% formic acid and loaded onto a Acclaim PepMap 100 trap column (75 µm × 20 mm, 100 Å, 3 µm particle size) and separated on a nanoACQUITY UPLC BEH130 C18 column (75 µm × 250 mm, 130 Å, 1.7 µm particle size), at a constant flow rate of 300 nL min-1, with a column temperature of 50 °C and a linear gradient of 2−60% acetonitrile/0.1% formic acid in 20 min, and then 60-98% acetonitrile/0.1% formic acid in 5 min, and then held isocratically for another 5 min. For mass spectrometer, it was operated under data-dependent acquisition (DDA), one scan cycle comprised of a full scan MS survey spectrum, followed by up to 12 sequential HCD MS/MS on the most intense signals above a threshold of 1e4. Full-scan MS spectra (600–2000 m/z) were acquired in the FT-Orbitrap at a resolution of 70,000 at 400 m/z, while HCD MS/MS spectra were recorded in the FT-Orbitrap at a resolution of 35,000 at 400 m/z. HCD was performed with a target value of 1e5 and normalization collision energy 25 was applied. AGC target values were 5e5 for full FTMS. For all experiments, dynamic exclusion was used with a single repeat count, 15 s repeat duration, and 30 s exclusion duration. There was one clean run between samples.

Analytical chemistry and chemical compounds

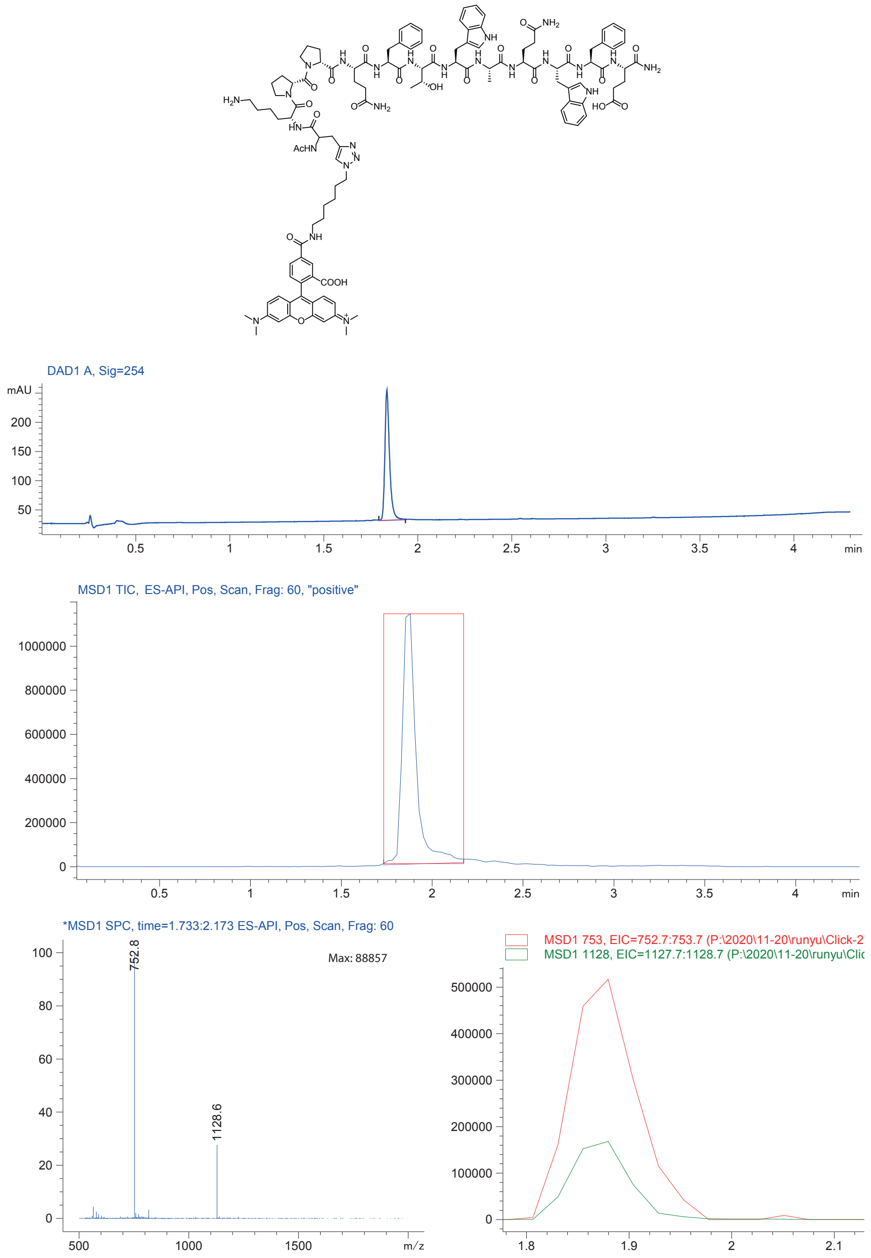

Structure and LC-MS QC data for the synthetic peptide WEHI-1881196.

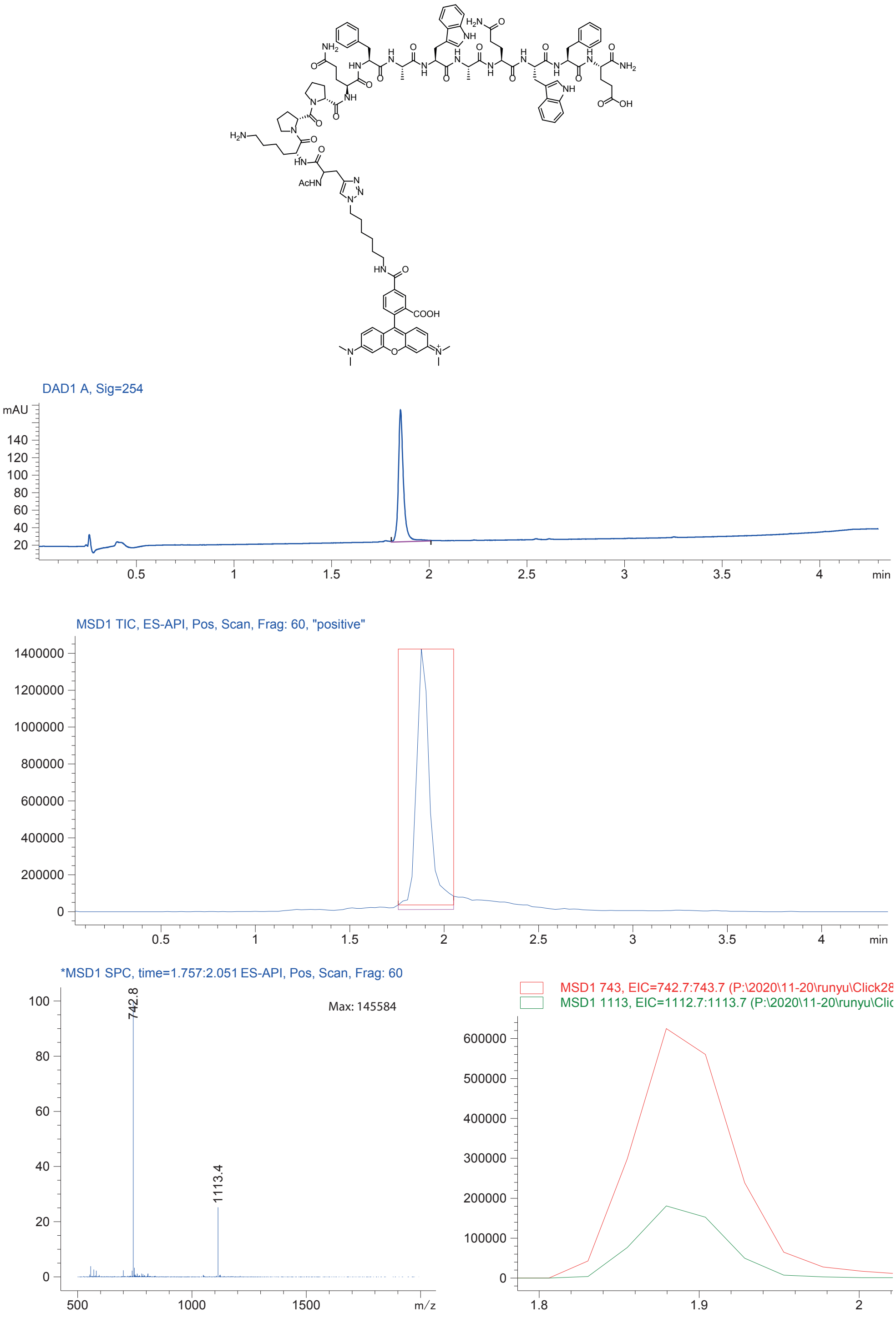

Structure and LC-MS QC data for the synthetic peptide WEHI-1881197.

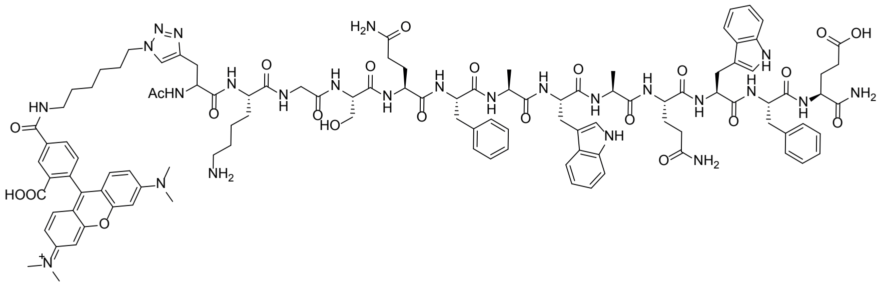

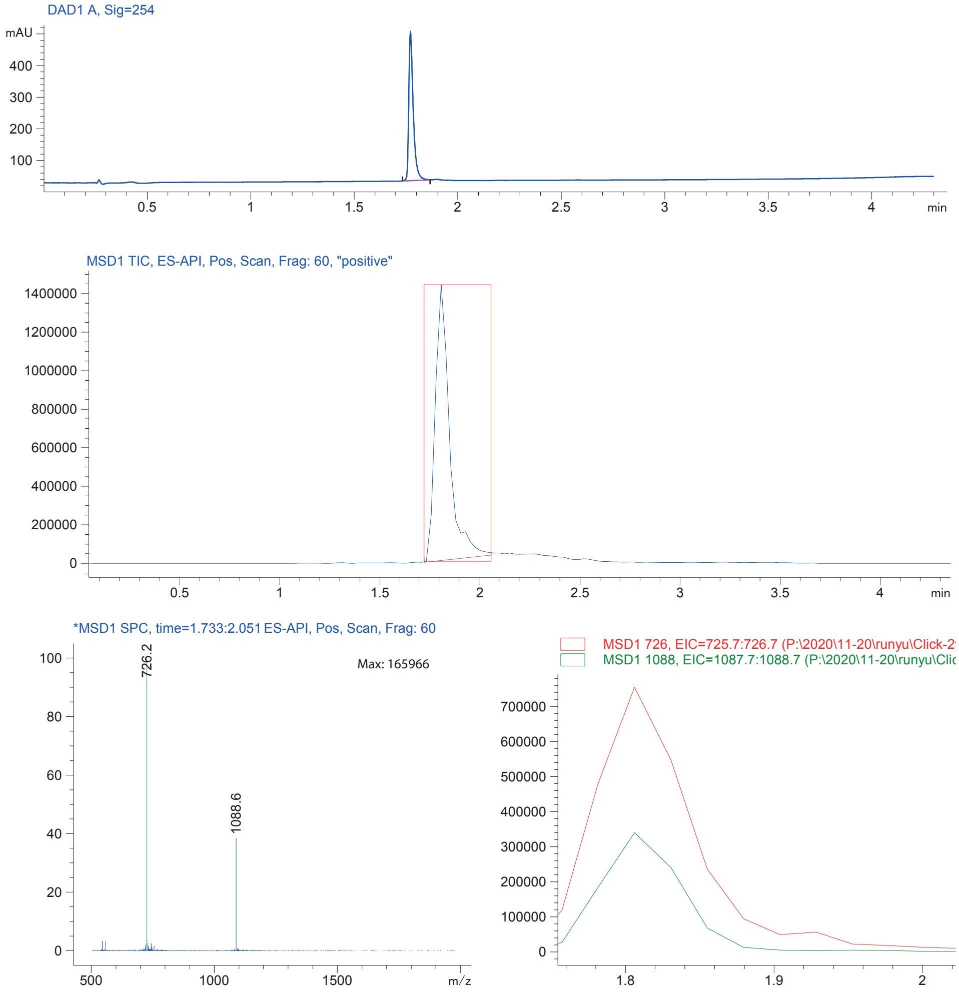

Structure and LC-MS QC data for the synthetic peptide WEHI-1881198.

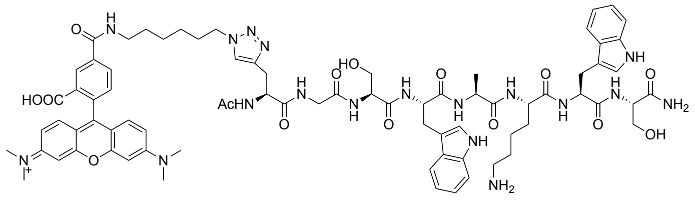

Ac-Ppg(TAMRA)-Gly-Ser-Trp-Ala-Lys-Trp-Ser-NH_2
WEHI-1886494

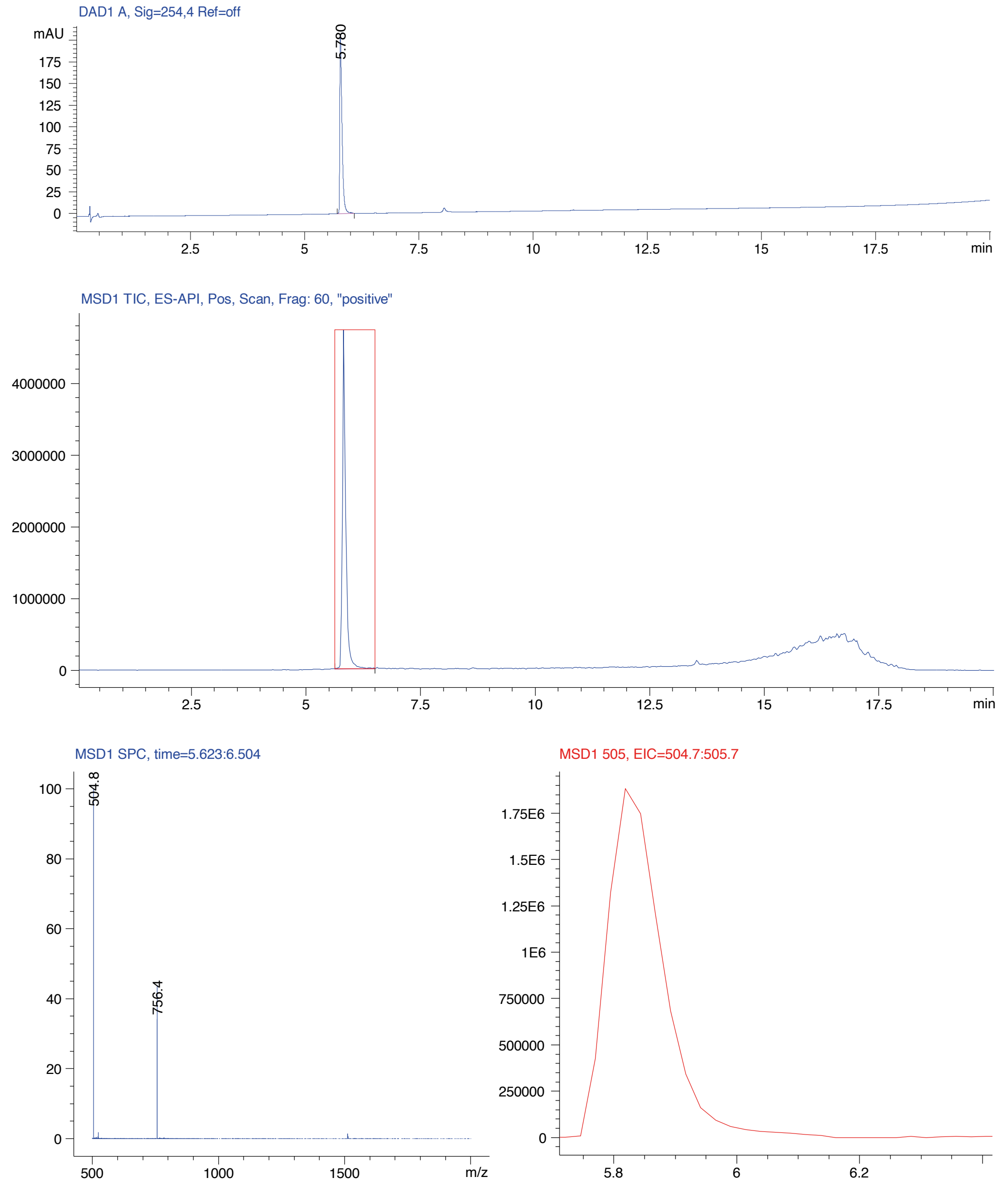

Structure and LC-MS QC data for the synthetic peptide WEHI-1886494.

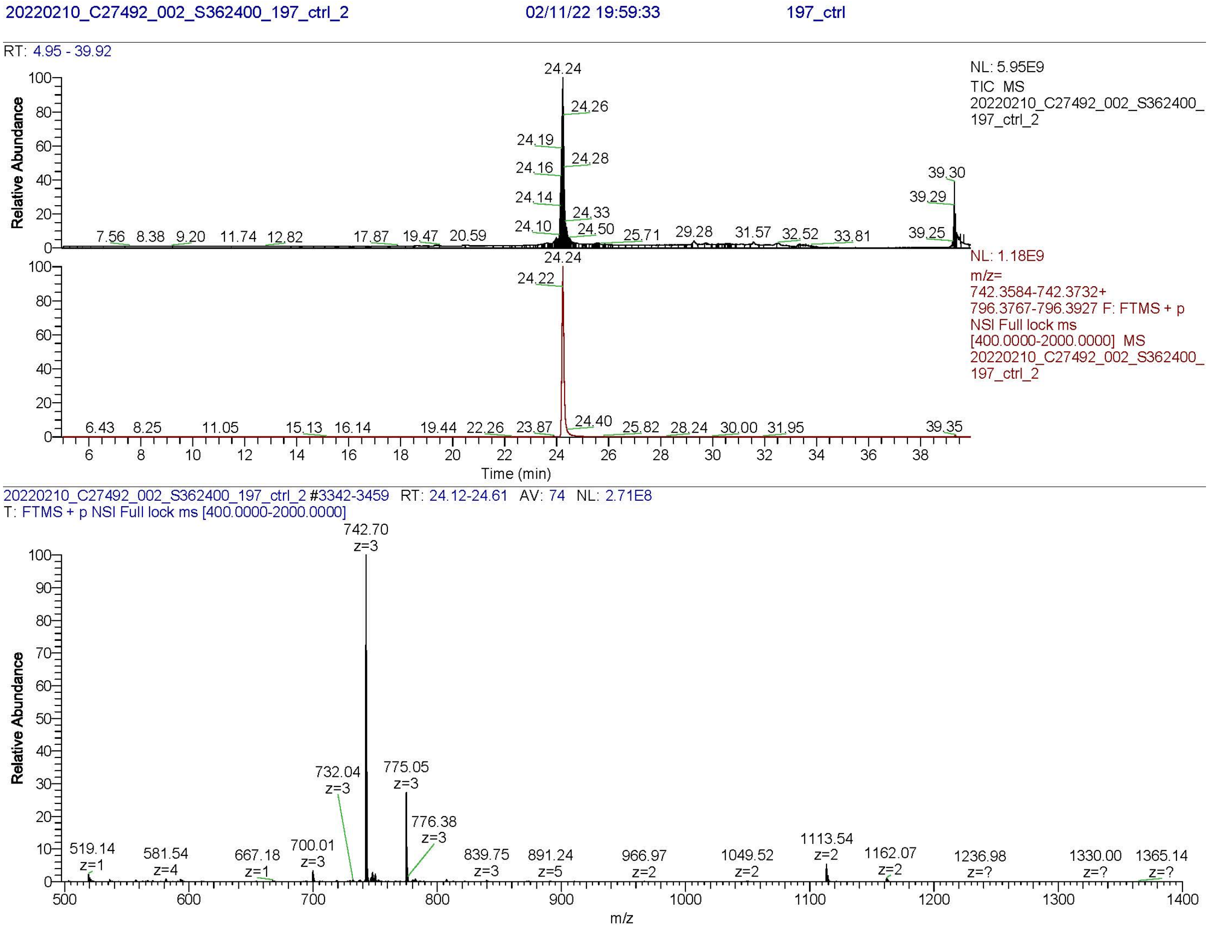

LC-MS data for glycopeptides from *Ce*DPY19 *in vitro* activity assays: WEHI-1881197 control.

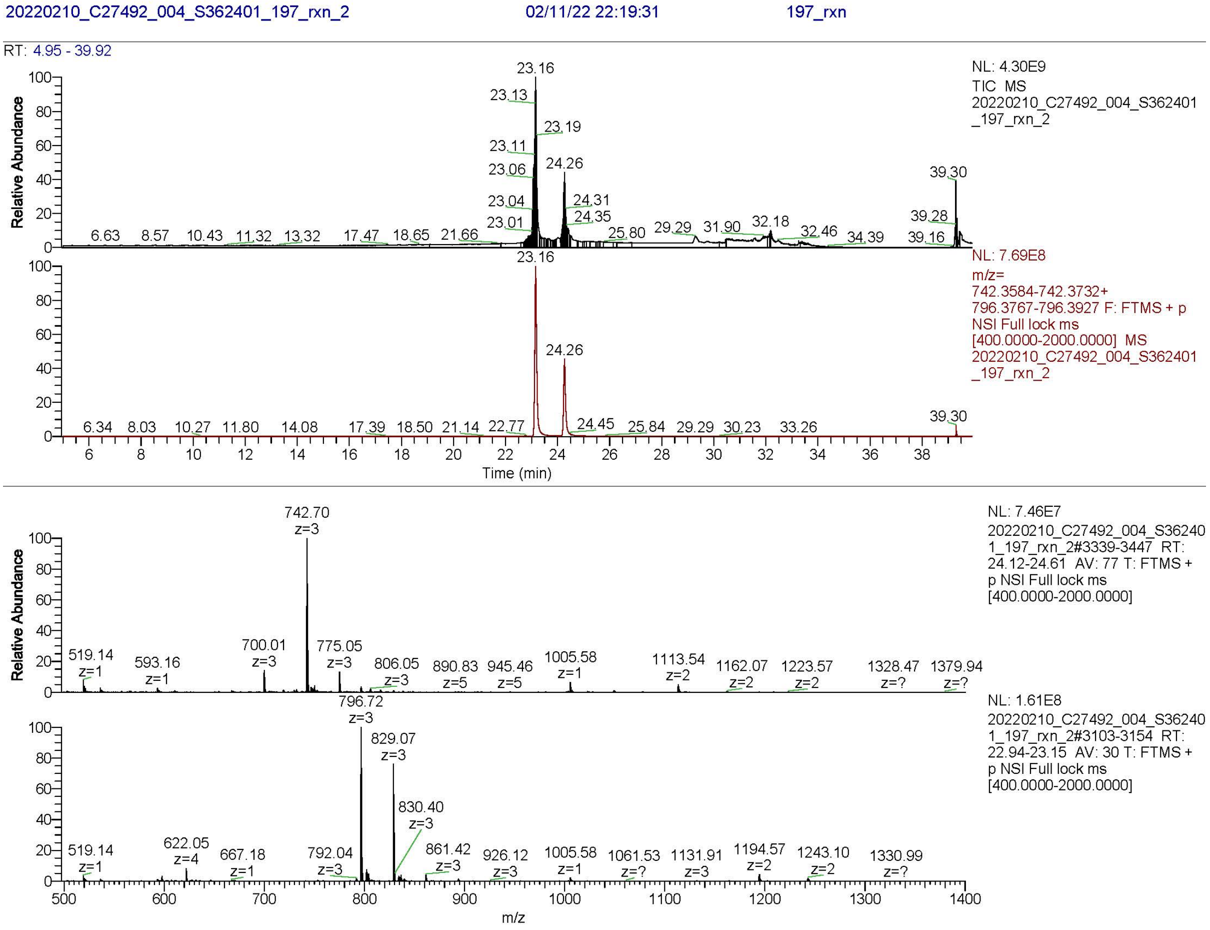

LC-MS data for glycopeptides from *Ce*DPY19 *in vitro* activity assays: WEHI-1881197 *Ce*DPY19 reaction.

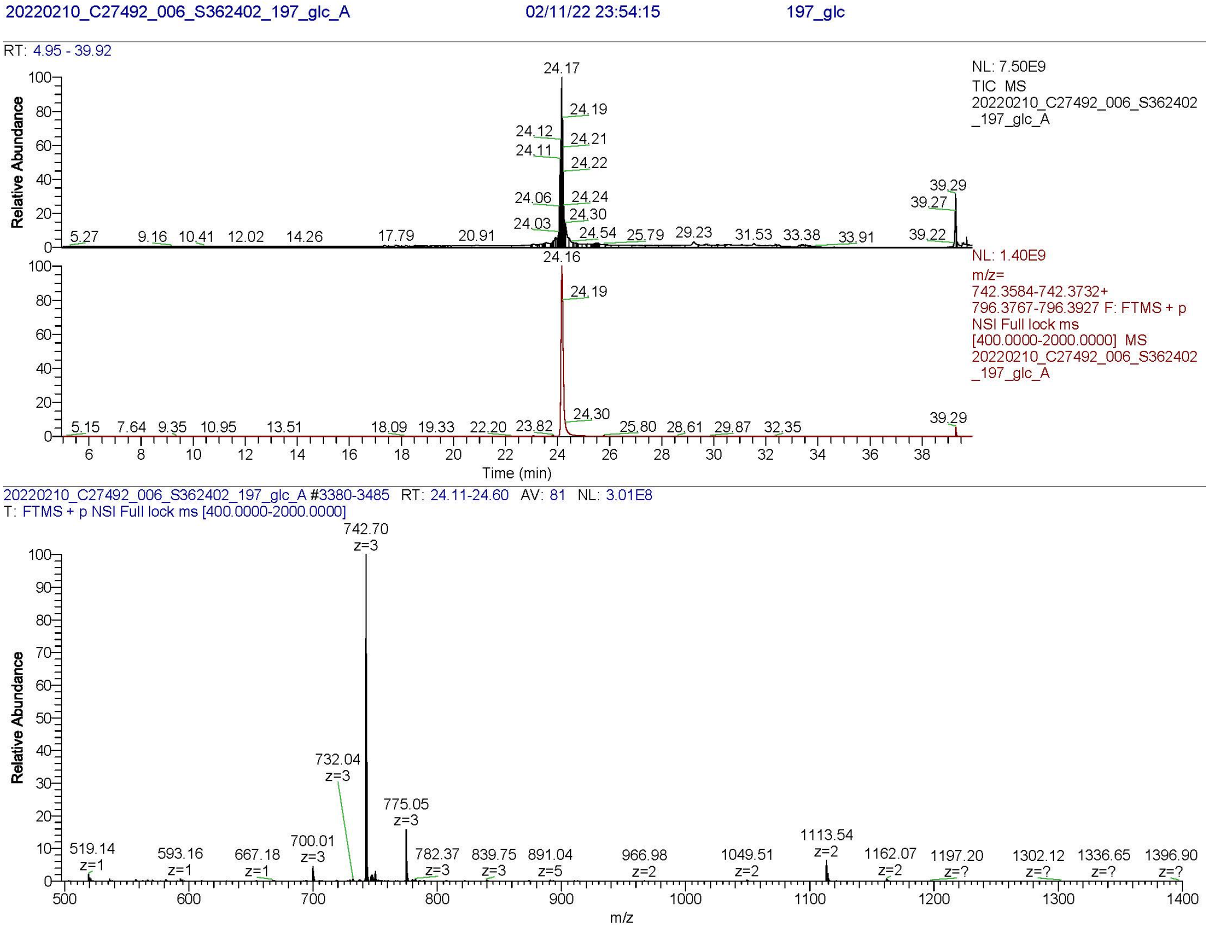

LC-MS data for glycopeptides from *Ce*DPY19 *in vitro* activity assays: WEHI-1881197 *Ce*DPY19 reaction with Dol25-P-Glc.

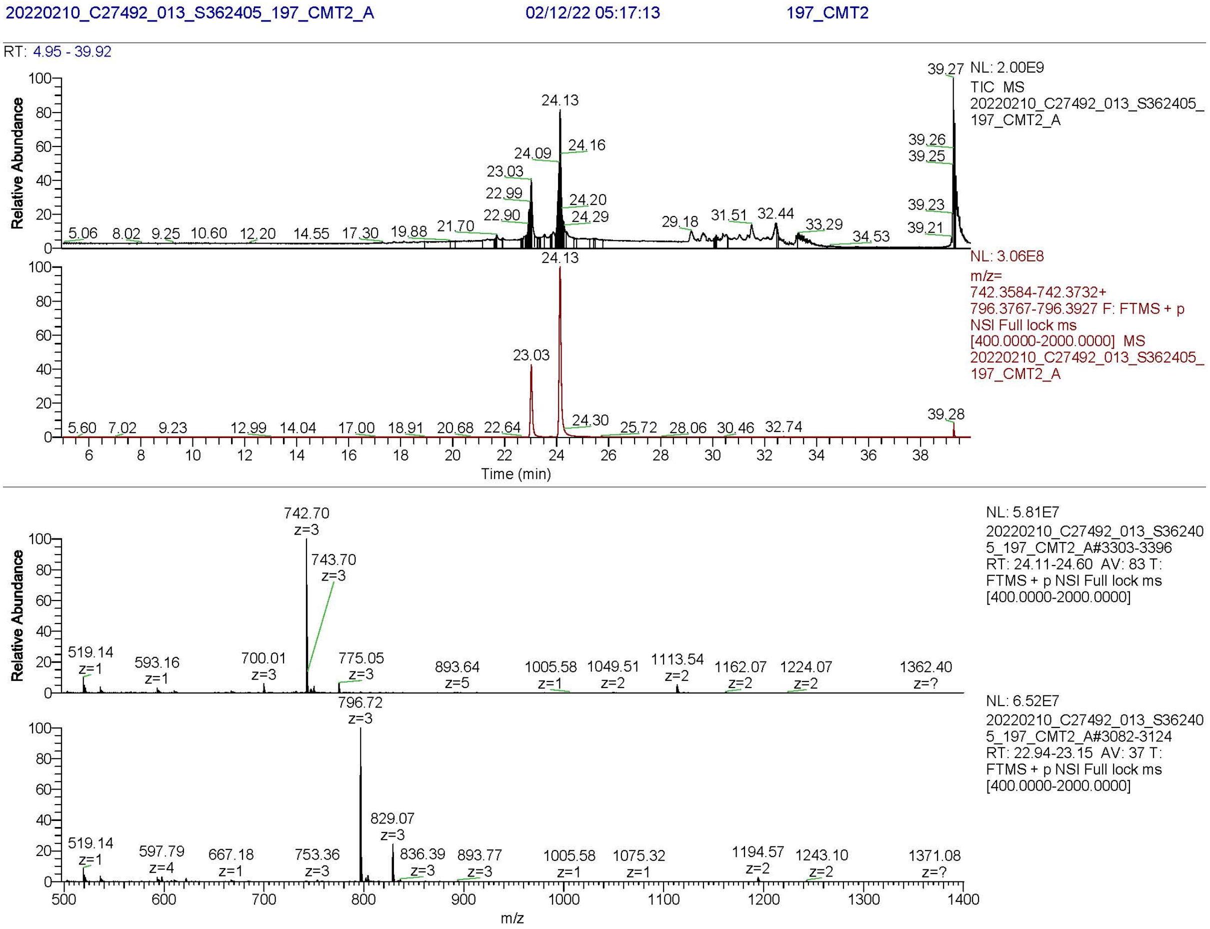

LC-MS data for glycopeptides from *Ce*DPY19 *in vitro* activity assays: WEHI-1881197 *Ce*DPY19 reaction in presence of CMT2-Fab.

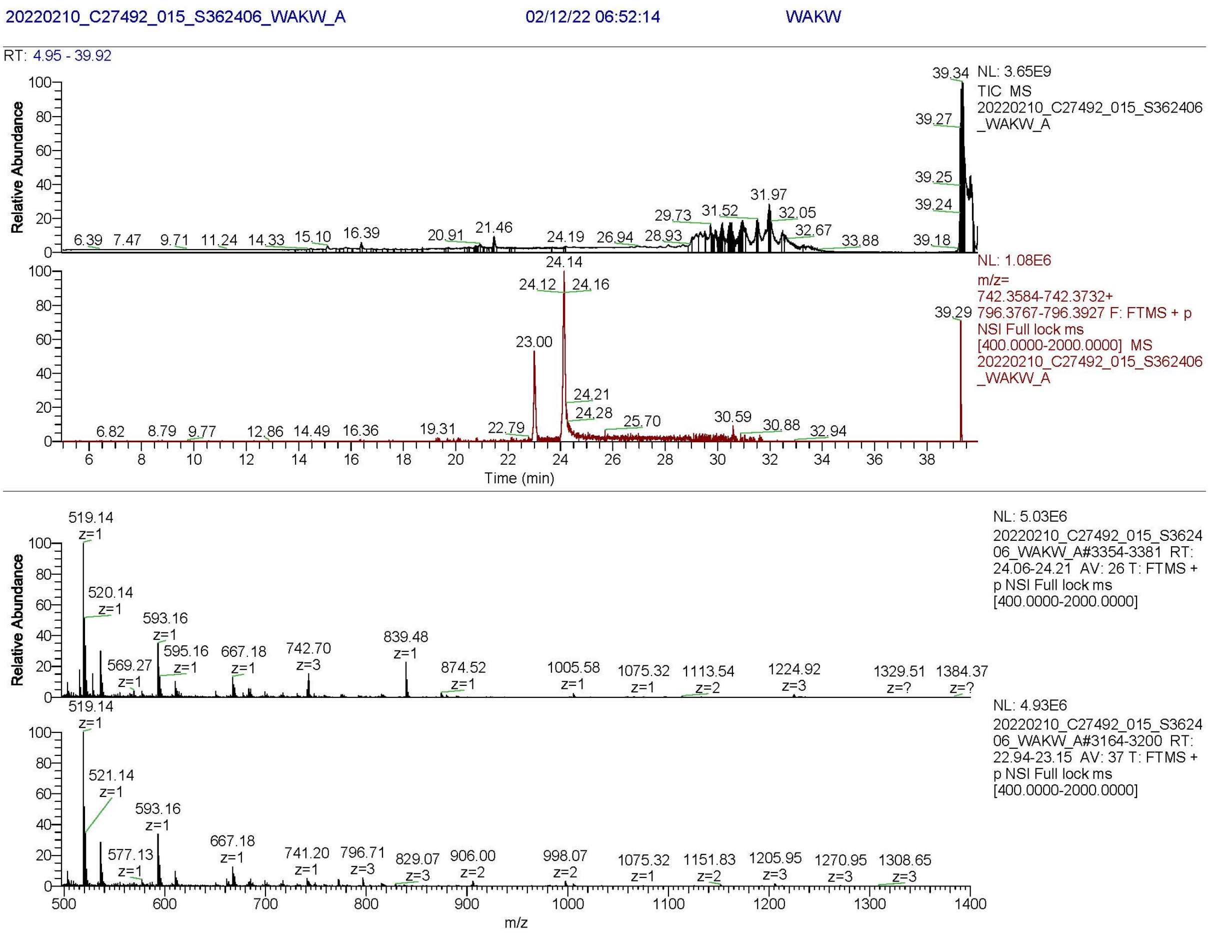

LC-MS data for glycopeptides from *Ce*DPY19 *in vitro* activity assays: TAMRA-Ac-Pra-GSWAKWS-NH$_2$ *Ce*DPY19 reaction.

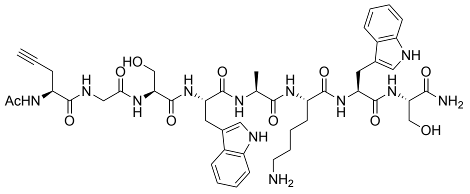

Ac-Ppg-Gly-Ser-Trp-Ala-Lys-Trp-Ser-NH$_2$
WEHI-1886493

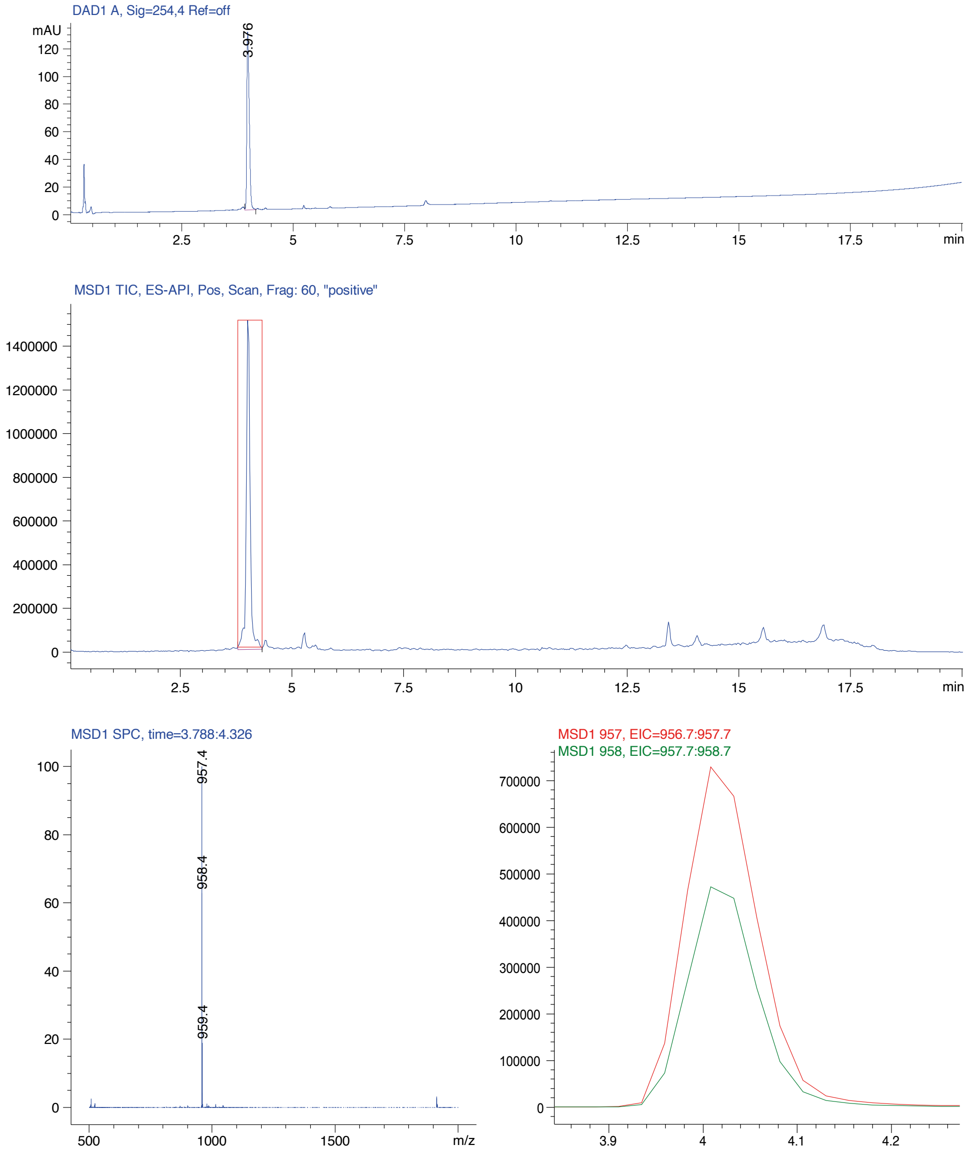

Structure and LC-MS QC data for the synthetic peptide WEHI-1886493.

Source Data for Supplementary Figure 2: Raw and uncropped Western blots.

E579A-Q

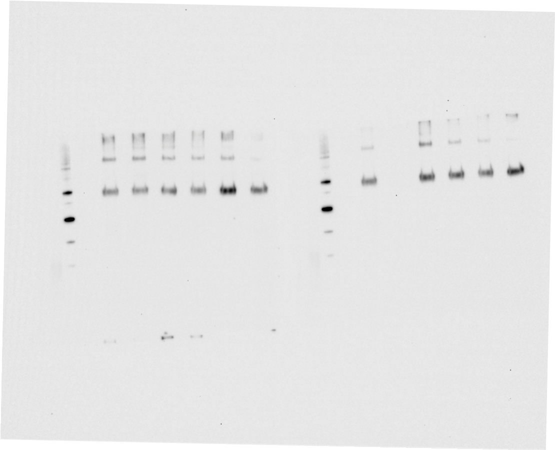

F401Y, R471A,Q, L472F, K473

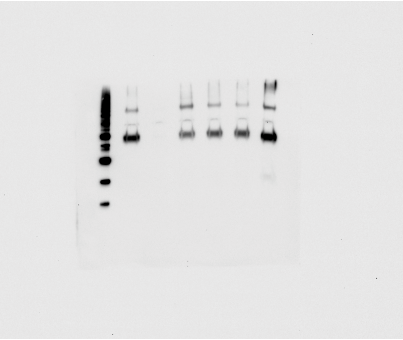

K473R,F, L474Y, H577A,Q,N

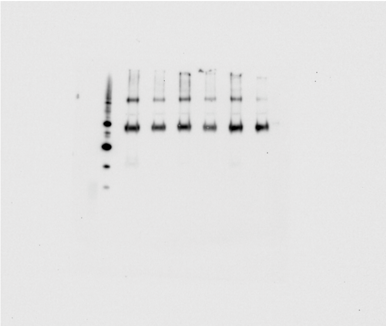

Y75A, F264A,M, E400A,Q, F401A

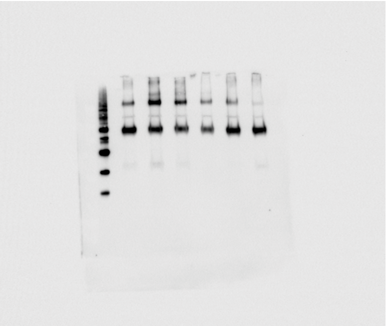

R69AK, E71AQDLM, Y75F, R211A, E220AQ, Q263A

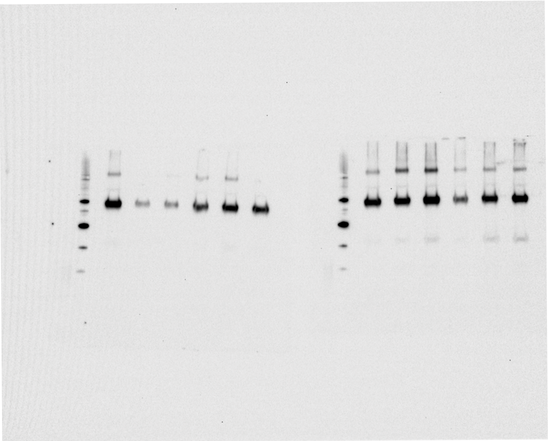

Supplement: Supplementary file 1 — Supplementary Figs. 1 and 2, Tables 1–3, Methods and Data. [file 41589_2022_1219_MOESM1_ESM.pdf]
