# Supplementary material for: Structure, sequon recognition and mechanism of tryptophan C-mannosyltransferase
Source: Nat Chem Biol. 2023 Jan 5;19(5):575–84. doi: 10.1038/s41589-022-01219-9 (PMC10154233; doi:10.1038/s41589-022-01219-9)
Supplement: Supplementary file 2 — Reporting Summary [file 41589_2022_1219_MOESM2_ESM.pdf]

nature research

Corresponding author(s): Kaspar P. Locher
Ethan D. Goddard-Borger

Last updated by author(s): Oct 17, 2022

Reporting Summary

Nature Research wishes to improve the reproducibility of the work that we publish. This form provides structure for consistency and transparency in reporting. For further information on Nature Research policies, see our Editorial Policies and the Editorial Policy Checklist.

Statistics

For all statistical analyses, confirm that the following items are present in the figure legend, table legend, main text, or Methods section.

n/a	Confirmed	
☐	☒	The exact sample size (*n*) for each experimental group/condition, given as a discrete number and unit of measurement
☐	☒	A statement on whether measurements were taken from distinct samples or whether the same sample was measured repeatedly
☐	☒	The statistical test(s) used AND whether they are one- or two-sided<br>*Only common tests should be described solely by name; describe more complex techniques in the Methods section.*
☒	☐	A description of all covariates tested
☒	☐	A description of any assumptions or corrections, such as tests of normality and adjustment for multiple comparisons
☐	☒	A full description of the statistical parameters including central tendency (e.g. means) or other basic estimates (e.g. regression coefficient) AND variation (e.g. standard deviation) or associated estimates of uncertainty (e.g. confidence intervals)
☒	☐	For null hypothesis testing, the test statistic (e.g. *F*, *t*, *r*) with confidence intervals, effect sizes, degrees of freedom and *P* value noted<br>*Give P values as exact values whenever suitable.*
☒	☐	For Bayesian analysis, information on the choice of priors and Markov chain Monte Carlo settings
☒	☐	For hierarchical and complex designs, identification of the appropriate level for tests and full reporting of outcomes
☒	☐	Estimates of effect sizes (e.g. Cohen's *d*, Pearson's *r*), indicating how they were calculated

Our web collection on statistics for biologists contains articles on many of the points above.

Software and code

Policy information about availability of computer code

Data collection	EPU2
Data analysis	MotionCor2, RELION 3.1, Gctf, CryoSPARC v3.2, Prism 9, Coot 8.9, PHENIX-1.17.1-3660, MolProbity, eLBOW, PyMol 2.5.2, UCSF Chimera, UCSF ChimeraX, Clustal Omega, Jalview

For manuscripts utilizing custom algorithms or software that are central to the research but not yet described in published literature, software must be made available to editors and reviewers. We strongly encourage code deposition in a community repository (e.g. GitHub). See the Nature Research guidelines for submitting code & software for further information.

Data

Policy information about availability of data

All manuscripts must include a data availability statement. This statement should provide the following information, where applicable:

- Accession codes, unique identifiers, or web links for publicly available datasets
- A list of figures that have associated raw data
- A description of any restrictions on data availability

Atomic coordinates of the CeDPY19 models have been deposited in RCSB Protein Data Bank (PDB) under accession numbers 7ZLH (apo), 7ZLH (peptide-bound), 7ZLI (Dol25-P-Man- bound) and 7ZLJ (Dol25-P-C-Man- and peptide-bound). The three-dimensional cryo-EM maps were deposited in the Electron Microscopy Data Bank (EMDB) under accession numbers EMD-14780 (apo), EMD-14779 (peptide-bound), EMD-14781 (Dol25-P-Man-bound), and EMD-14782 (Dol25-P-C-Man- and peptide-bound). MS data to quantitate tryptophan C- mannosyaltion on RNAse2 has been deposited to the PRIDE proteomics repository under the accession number: PXD032391 using the Username: reviewer_pxd032391@ebi.ac.uk and Password: sItWEoNT.

Field-specific reporting

Please select the one below that is the best fit for your research. If you are not sure, read the appropriate sections before making your selection.

☒ Life sciences ☐ Behavioural & social sciences ☐ Ecological, evolutionary & environmental sciences

For a reference copy of the document with all sections, see nature.com/documents/nr-reporting-summary-flat.pdf

Life sciences study design

All studies must disclose on these points even when the disclosure is negative.

Sample size	The sample sized varied between n=1-3 for in vivo and in vitro experiments. For experiments with sample size equal 1, complementary similar experiments were performed to ensure their integrity. For all structures thousands of micrography were recorded and were inspected for homogeneity within the relevant datasets. Further, cryo-EM structures at high resolution can only be calculated if the respective particles are homogeneous in their structure and data quality.
Data exclusions	No data was excluded in this study.
Replication	All replicates were successful.
Randomization	Randomization is not relevant to the biochemical and structural experiments reported in this study because there is no opportunity for the introduction of human bias in the generation or interpretation of the data.
Blinding	Randomization is not relevant to the biochemical and structural experiments reported in this study because there is no opportunity for the introduction of human bias in the generation or interpretation of the data.

Reporting for specific materials, systems and methods

We require information from authors about some types of materials, experimental systems and methods used in many studies. Here, indicate whether each material, system or method listed is relevant to your study. If you are not sure if a list item applies to your research, read the appropriate section before selecting a response.

Materials & experimental systems

n/a	Involved in the study	
☐	☒	Antibodies
☐	☒	Eukaryotic cell lines
☒	☐	Palaeontology and archaeology
☒	☐	Animals and other organisms
☒	☐	Human research participants
☒	☐	Clinical data
☒	☐	Dual use research of concern

Methods

n/a	Involved in the study	
☒	☐	ChIP-seq
☒	☐	Flow cytometry
☒	☐	MRI-based neuroimaging

Antibodies

Antibodies used	Fab-CMT2, produced in house, described in this publication, anti-Flag mouse IgG1 (SigmaAldrich, F3165), mouse anti-M13 monoclonal antibody (cat: 27-9420-01, GE Healthcare), goat anti-mouse horseradish peroxidase conjugate (1:10000, ThermoFisher, 62-6520)
Validation	We describe biophysical and structural characterization of Fab-CMT2 in this paper. The used commercial antibodies were validated by the respective manufacturer.

Eukaryotic cell lines

Policy information about cell lines

Cell line source(s)	Sf9 (ATCC® CRL-1711™)
Authentication	The cells were purchased directly from the manufacture. They were not authenticated by us.
Mycoplasma contamination	The cells were not tested for mycoplasma.
Commonly misidentified lines (See ICLAC register)	No commonly misidentified cells were used in this study.
