# Supplementary figures and images for: Structure, sequon recognition and mechanism of tryptophan C-mannosyltransferase
Source: Nat Chem Biol. 2023 Jan 5;19(5):575–84. doi: 10.1038/s41589-022-01219-9 (PMC10154233; doi:10.1038/s41589-022-01219-9)

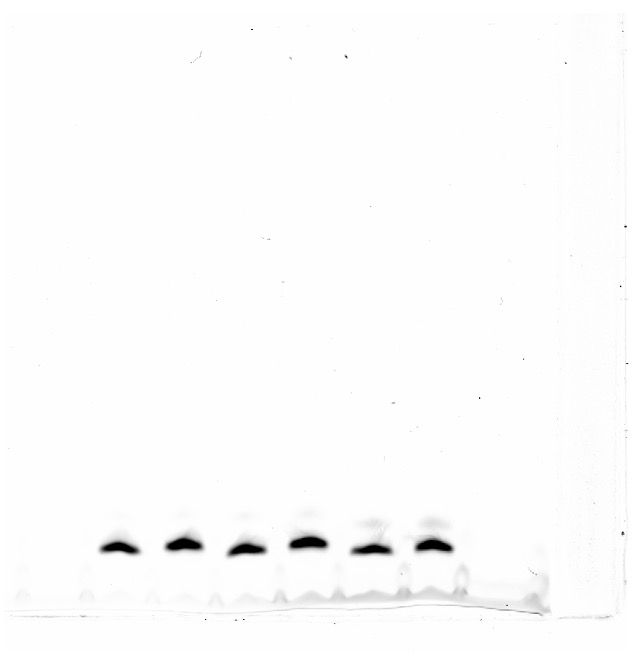

Supplement: Source Data Fig. 1 — Unprocessed Tricine–SDS–PAGE, raw data of chromatogram and unprocessed Tricine–SDS–PAGE. [file 41589_2022_1219_MOESM3_ESM.zip › Source_Data_Fig1/Fig1b_Extended_Data_Figure_1a_Tricine-SDS-PAGE.jpg]

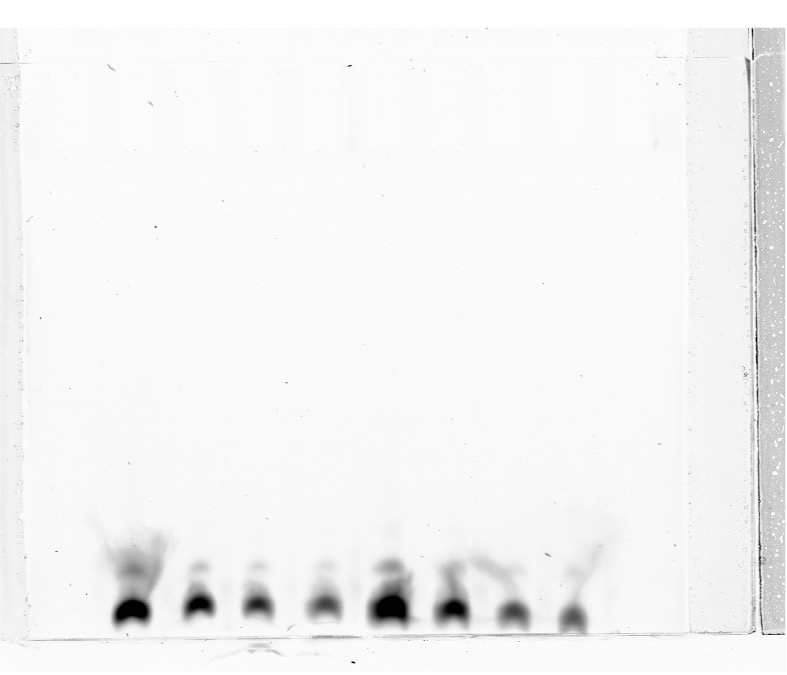

Supplement: Source Data Fig. 1 — Unprocessed Tricine–SDS–PAGE, raw data of chromatogram and unprocessed Tricine–SDS–PAGE. [file 41589_2022_1219_MOESM3_ESM.zip › Source_Data_Fig1/Fig1d_Tricine-SDS-PAGE.jpg]

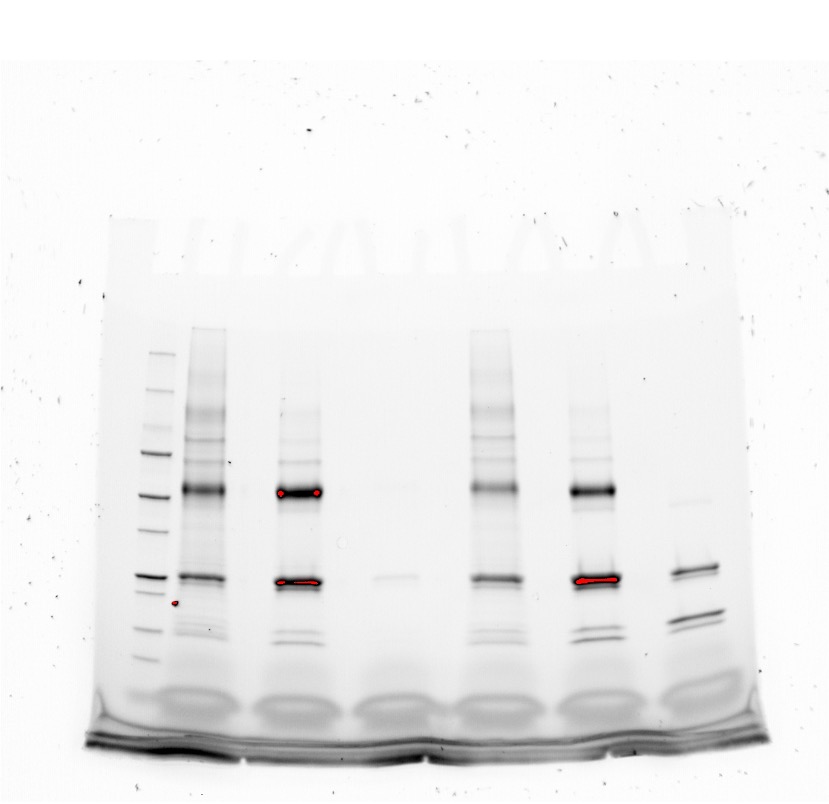

Supplement: Source Data Extended Data Fig. 2 — Raw data of chromatogram, unprocessed SDS–PAGE and input data of graph. [file 41589_2022_1219_MOESM6_ESM.zip › Source_Data_Extended_Data_Figure_2/Extended_Data_Figure_2b_SDS-PAGE.jpg]
